# Bioavailability of and US Infant Exposure to Arsenic, Cadmium, Lead, Mercury, and Per- and Polyfluoroalkyls from Human Milk and Infant Formula: Results from a Series of Systematic Reviews

**DOI:** 10.1016/j.advnut.2026.100628

**Published:** 2026-04-09

**Authors:** Lauren E O’Connor, Rachel C Thoerig, Cassi N Leta, Arin A Balalian, Shailesh M Advani, Trish Bosse, Margaret J Foster, Kyle M Holland, Kathryn G Dewey, Mandy M Fisher, Aubrey L Galusha, Carin A Huset, Amanda J MacFarlane, Maureen K Spill

**Affiliations:** 1 Agriculture, Food, and Nutrition Evidence Center, Texas A&M AgriLife Research, Fort Worth, TX, United States; 2 Center for Systematic Reviews and Research Syntheses, Medical Sciences Library, Texas A&M University, College Station, TX, United States; 3 Department of Nutrition, University of California-Davis, Davis, CA, United States; 4 Environmental Health Science and Research Bureau, Health Canada, Ottawa, ON, Canada; 5 New York State Department of Health, Biggs Laboratory, Wadsworth Center, Albany, NY, United States; 6 Department of Environmental Health Sciences, College of Integrated Sciences, University at Albany, Albany, NY, United States; 7 Minnesota Department of Health, Public Health Laboratory, Saint Paul, MN, United States; 8 Department of Nutrition, Texas A&M University, College Station, TX, United States

**Keywords:** infant toxicology, public health, trace elements, heavy metals, bottle feeding, lactation

## Abstract

Infants may be exposed to contaminants from environmental and food sources, including human milk (HM) or infant formula (IF). The objective of this series of systematic reviews (PROSPERO: CRD42024530332, CRD42024530336, CRD42024530339, and CRD42024530344) was to *1*) assess infant exposure to contaminants from HM and/or IF in the United States and *2*) assess the bioavailability of these contaminants from HM and IF when consumed by infants. The protocol was developed with a technical expert panel (TEP). Through 2 April, 2025, CAB Abstracts, CENTRAL, CINAHL, Embase, and MEDLINE databases were searched for peer-reviewed articles published in English. Another TEP critically appraised sample collection and contaminant assessment methods for included articles. Studies needed to report contaminant concentrations of biospecimens based on infant feeding (HM only, IF only, or HM and IF). Results were narratively described. Risk of bias was assessed using ROBINS-E. From 6799 unique records, 7 articles from 4 studies were identified. The New Hampshire birth cohort reported infant urinary concentrations of arsenic (*n* = 4 articles), cadmium (*n* = 2), lead (*n* = 1), and mercury (*n* = 1) between 2009 and 2019. Three other studies reported infant blood lead concentrations between 1975 and 1994. All articles had acceptable contaminant assessment methods, and most (*n* = 6 articles) had low risk or some concerns of bias. Collectively, most evidence was from 1 cohort or published 30+ y ago and lacked demographic and geographic diversity. Therefore, conclusions could not be made about exposure to arsenic, cadmium, lead, or mercury from HM and/or IF for infants in the United States. No evidence was found that reported on per- and polyfluoroalkyl substances in infant biospecimens or bioavailability of any of the contaminants from HM or IF. These findings highlight the need for research about infant exposure to and bioavailability of arsenic, cadmium, lead, mercury, and per- and polyfluoroalkyl substances from HM or IF in the United States.

**Statements of significance**

This series of systematic reviews highlights critical research gaps about US infant exposure to and bioavailability of arsenic, cadmium, lead, mercury, and per- and polyfluoroalkyl substances from human milk and/or infant formula. The paucity of evidence underscores the need for biomonitoring or national surveillance to understand US infant exposure to potential contaminants in human milk and infant formula to inform feeding practice guidelines.

## Introduction

Environmental contaminants found in foods, such as arsenic, cadmium, lead, mercury, or per- and polyfluoroalkyl substances (PFAS), can have consequences for all people [1], particularly infants [2]. Infants develop rapidly, requiring high energy and nutrient intakes from food relative to their body size; this high ratio of food intake to body weight, in addition to high metabolic rates, makes them vulnerable to contaminants in foods [[3], [4], [5]]. Other characteristics that may increase vulnerability to contaminant exposure in infants include: faster breathing rates [4], higher surface area for organs [5], immature immune and excretion systems [3,6], and faster resting metabolic rates during infancy compared with adulthood [3,5]. Assessing exposure to these contaminants from all sources, including foods, allows for investigation of associations with health and developmental outcomes for infants [[7], [8], [9], [10], [11], [12]].

During the first year of life, infants primarily consume human milk (HM) and/or infant formula (IF), with complementary foods (including water) introduced around 6 mo [13]. Although infants can be exposed to contaminants via several sources, HM and IF can be potential contributors to total exposure. Evidence from systematic reviews (SRs) suggests that higher lead, mercury, and PFAS [specifically, perfluorooctanoic acid (PFOA) and perfluorooctanesulfonic acid (PFOS)] exposure during pregnancy or lactation correlated with higher concentrations of those contaminants in HM [14]. Further, PFOA and PFOS were detected in HM in the United States based on data collected from 2008 to 2023 [15]. Heavy metals, specifically arsenic, cadmium, lead, and mercury, were also detected in HM as well as in IF, though much of the data were collected prior to 2000 [15] and may not reflect current exposures or the modern-day food supply. In addition to contaminant concentrations of HM and IF, exposure estimates also need to consider the amount consumed [16] as HM and IF are the predominant foods consumed during infancy.

In addition to the amount consumed, understanding bioavailability of potential contaminants in HM and IF can inform infant exposure estimates. For example, PFAS can be detected in HM [17], but whether or how PFAS are absorbed and metabolized by the infant and subsequent health effects are unclear [18]. In contrast, research shows that infants absorb ≤50% of lead from food [19,20]. Contaminants in foods may also interfere with the absorption, digestion, metabolism, and excretion of essential nutrients. For example, lead competes with calcium for binding sites in bone [21,22] and mercury and cadmium disrupt calcium and iron transport [23,24], which negatively impacts bone development and contributes to iron deficiency anemia. Some nutrients may also be protective by reducing contaminant absorption and metabolism and promoting excretion. For example, selenium binds to mercury [25], adequate iron status reduces lead absorption [26], and folic acid, which is fortified in many staple foods, increases methylation of inorganic arsenic and facilitates urinary excretion [27,28]. Collectively, this exemplifies how the unique food matrix of HM or IF may affect the interrelated availability, absorption, and metabolism of contaminants and essential nutrients.

Currently, no routine, nationally representative biomonitoring of arsenic, cadmium, lead, mercury, and PFAS exposure in infants is conducted across the United States. To inform estimates of exposure to contaminants from HM and/or IF, we conducted SRs to: *1*) assess infant exposure to contaminants from HM and/or IF in the United States from studies that measured contaminants in infant biospecimens in relation to the consumption of HM and/or IF, and *2*) assess the bioavailability of these contaminants from HM and IF when consumed by infants from countries rated “high” or “very high” on the human development index [29].

## Methods

**FIGURE 1:**
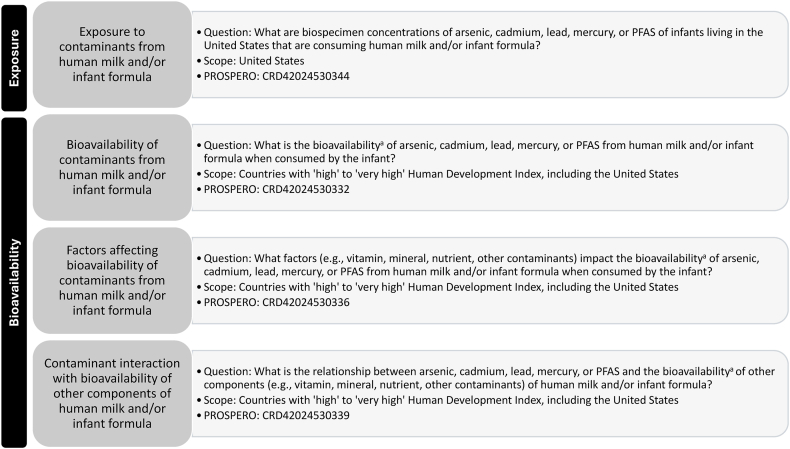
Research questions addressed in this systematic review. PFAS, per- and polyfluoroalkyl substances. ^a^Bioavailability was defined as the ratio of contaminant concentrations in human milk or infant formula being consumed by the infant to contaminant concentrations in infant biospecimens.

This research was part of a series of SRs (Supplemental Table 1) commissioned by the US Food and Drug Administration’s human foods program within its mission area of food chemical safety [30]. The SRs were designed to identify and synthesize peer-reviewed scientific literature about infant exposure to arsenic, cadmium, lead, mercury, and PFAS from HM and/or IF in the United States (Supplemental Table 1). Four SRs are presented in this manuscript (Figure 1; PROSPERO: CRD42024530332, CRD42024530336, CRD42024530339, and CRD42024530344), and the others have been published [14,15].

The protocols, including the review questions, search strategy, inclusion/exclusion criteria (including the operational definition of bioavailability), risk of bias (ROB) assessment, plan for synthesis and investigation of heterogeneity, were developed in collaboration with a technical expert panel (TEP) with experience in human biomonitoring, infant nutrition, and HM (KGD and MMF) prior to review commencement. The protocols were registered a priori in PROSPERO. We adhered to the PRISMA guidelines [31] (Supplemental Appendix A) and met specifications of a high-quality SR according to a measurement tool to assess systematic reviews (AMSTAR 2) critical appraisal tool [32] (Supplemental Appendix B).

### Search strategy

Two SR information specialists (MJF and KMH) developed the search strategy, and a third conducted a peer review (SG). The strategy informed multiple research questions about infant exposure to contaminants from HM and/or IF (Supplemental Table 1), including 1 of the previous publications, which was about contaminant concentrations in HM and IF in the United States [15]. The 2 search concepts included search strings for *1*) HM, IF, and lactation, and *2*) arsenic, cadmium, lead, mercury, or PFAS. Each concept was built by combining synonyms and relevant thesaurus terms from each database. The PFAS concept was built with terms found in another SR [33]. The search was developed in MEDLINE (Ovid) and then was translated for the other 4 databases: CAB Abstracts (Ovid), Embase (Ovid), CENTRAL (Cochrane Library), and CINAHL (EBSCO). These databases were searched for peer-reviewed articles published in English, with no restrictions on publication date, up to 2 April, 2025 (Supplemental Table 2). Also, a keyword manual search of the *Journal of Environmental Exposure Assessment* was completed based on the TEP’s recognition of the journal’s relevance and omission from indexed databases. Additionally, 2 reviewers independently conducted backward citation searches to identify pertinent titles cited in the reference list of included articles.

We also conducted a post hoc manual search of citations related to the health outcomes and measures of the environment (HOME) study. This study was not originally captured in our database search because most HOME articles were indexed with key terms related to pregnancy, which were out of scope. To expand our search strategy, we manually searched references cited in the clinicaltrials.gov registry of the HOME study [34], the list of publications from the HOME study website [35], as well as the Rochester lead-in-dust study website [36], which was identified during the manual search of the HOME study.

### Inclusion and exclusion criteria

Detailed inclusion and exclusion criteria for the SR about exposure to contaminants from HM and/or IF for infants living in the United States are described in Supplemental Table 3. Detailed inclusion and exclusion criteria for the 3 SRs about bioavailability of contaminants from HM and IF are described in Supplemental Tables 4‒6.

The target population was general US infants consuming HM and/or IF. Populations were eligible if the infants were aged ≤12 mo. For example, an infant born on 5 June, 2025, would be eligible for inclusion until their first birthday on 5 June, 2026. This age was selected because any HM consumption steadily decreases during the first 12 mo of life in the United States, from 83.2% at 0 mo to 35.9% at 12 mo [37]. It is also recommended by the US Centers for Disease Control and Prevention for infants to transition from IF to pasteurized cow milk or fortified unsweetened soy beverages by 12 mo [38]. Studies that exclusively enrolled infants who were born prematurely or diagnosed with a disease or developmental disorder, or hospitalized with an illness or injury, were out of scope and therefore excluded. Specialized guidelines would be needed for those groups to tailor to their specific needs.

For the bioavailability questions, the same population criteria were used but expanded beyond the United States to include infants aged ≤12 mo from all countries rated “high” or “very high” on the human development index [29]. This was because the pharmacokinetic processes of contaminant absorption, distribution, metabolism, or excretion that determine concentrations and bioavailability in the body [39] are unlikely to differ based on geographic location.

The outcome of interest was exposure to contaminants from HM or IF. This was operationalized as reported concentrations of total and inorganic arsenic, cadmium, lead, total mercury and methylmercury, or select legacy PFAS in biospecimens collected from infants in relation to their consumption of HM and/or IF. Thus, contaminant concentrations in biospecimens needed to be reported based on infant feeding (HM only, IF only, or HM and IF). We prioritized PFOA, PFOS, PFNA (perfluorononanoic acid), PFHxS (perfluorohexanesulfonic acid), and total PFAS of the >12,000 known PFAS chemicals. The selected legacy PFAS were chosen based on their established toxicological reference values for assessing food contamination, extensive research, and acceptable methods for analytical measurement [[40], [41], [42], [43]]. Cord blood, meconium, and hair collected at birth were deemed an ineligible outcome because collection would occur prior to infant exposure to HM and/or IF.

For the bioavailability SRs, the TEP informed the operational definition of bioavailability as the ratio of contaminant concentrations in HM and/or IF being consumed by infant to contaminant concentrations in infant biospecimens.

The TEP determined that eligible study designs included both randomized and nonrandomized designs: randomized controlled trials, nonrandomized controlled trials, quasi-experimental studies, prospective or retrospective cohort studies, cross-sectional studies, descriptive studies, and other designs without a comparison group (Supplemental Figure 1). For most questions related to bioavailability, descriptive studies and other designs without a comparison group were deemed ineligible because a comparator group was part of the analytical framework (Supplemental Figures 2‒4).

### Screening and data extraction

All articles were dually, independently screened using DistillerSR (Evidence Partners; 2020) with screening forms developed according to the inclusion and exclusion criteria (Supplemental Tables 3‒6). Screening forms were piloted on ≥25 articles. Given the overlap in exposures and/or outcomes across the research questions, articles were included during title/abstract screening if a contaminant of interest was measured in an infant biospecimen (e.g., blood, urine, and hair), HM, or IF. Full-text screening was separated into 2 phases. First, we extracted high-level data documenting the measured contaminant and source (infant biospecimen, HM, and/or IF), and data collection site (country). Then, we used Stata (StataCorp LLC) to sort the articles into SR question-specific datasets. During the second phase of full-text screening, we used the extracted data to confirm all inclusion criteria were met for each SR question.

Based on each research question, we extracted additional data related to the analytic framework (Supplemental Figures 1‒4) for included studies. Trained reviewers extracted data using a systematic approach and standardized forms that were piloted on 2‒3 articles to ensure consistency. One reviewer extracted qualitative data, including study characteristics, population characteristics, exposure source, analytic techniques, sample collection methods, reported or measured consumption of HM only, IF only, or a combination of HM and IF, and funding source. A second reviewer checked for accuracy and completeness. Two reviewers independently extracted quantitative data (including contaminant concentrations, sample size, and results of statistical analyses) and a third cross-checked for accuracy. Our study objective focused exclusively on peer-reviewed published literature; therefore, unpublished data were considered out of scope, and authors were not contacted.

### ROB

Two reviewers independently evaluated ROB according to study design using ROB 2 for randomized controlled trials [44], Risk of Bias In Non-randomized Studies of Interventions (ROBINS-I) [45,46], or the Risk Of Bias In Non-randomized Studies of Exposure (ROBINS-E) tool [47]. Supplemental Table 7 describes the operationalization of the tools used. ROB assessments were piloted on 2‒3 studies per study design included in the SR to ensure consistency.

Bias was assessed due to confounding, measurement of exposure and outcome, postexposure interventions, missing data, selection of participants into the study, and selective reporting of results. Domains were rated as low, some concerns, or high ROB. Domain-level discrepancies were resolved via discussion, including consultation with a third reviewer as needed. The highest ROB score across domains was applied as the overall rating. For the question related to exposure to contaminants from HM and/or IF of infants in the United States (Figure 1), ROB due to confounding, exposure assessment method, and postexposure intervention was not considered as sources of bias, as informed by the TEP in the analytical framework, due to the descriptive nature of this research question. Therefore, these domains were rated as low risk.

### Critical appraisal of contaminant assessment methods

Experts in human biomonitoring and environmental contaminant surveillance, particularly for heavy metals and PFAS, comprised our second TEP (ALG, CAH, and JLR). They reviewed the analytic methods used to measure contaminant concentrations and the appropriateness of biospecimen sample collection methods for all included studies. Articles were evaluated by 2 experts and categorized as “acceptable” or as having some concerns due to insufficient methodological or quality control reporting. A third expert adjudicated any disagreements.

### Data synthesis and post hoc assessment

Data syntheses were stratified by contaminant in relation to consumption of HM only, IF only, or HM and IF. Sources of heterogeneity (e.g., population characteristics, geographic location, and date of data collection) and TEP critical appraisal were described narratively and considered in the conclusions. For each conclusion, we conducted sensitivity analyses by excluding articles rated as high ROB.

We also conducted a post hoc assessment to summarize available evidence on associations between contaminant concentrations in HM or IF and contaminant concentrations in infant biospecimens rather than assessing bioavailability directly. The aim of this assessment was to summarize this evidence that may help inform future research priorities.

All extracted data are summarized in the manuscript or in the Supplemental Materials.

### Certainty of evidence

The certainty of evidence for each conclusion was evaluated using the grading of recommendations assessment, development, and evaluation (GRADE) [48] approach. This framework evaluates ROB, inconsistency, indirectness, imprecision, and publication bias. For observational studies, additional elements such as dose-response relationships, magnitude of effect, and residual confounding are considered applicable.

### Protocol deviations

We planned a priori to conduct random-effects meta-analyses. However, a small number of articles were identified in our search, most of which came from the same cohort. Therefore, we determined that meta-analyses were not appropriate, which also precluded our ability to statistically test for publication bias. We added CAB Abstracts as an additional database and manually searched the HOME study and Rochester lead-in-dust study, as described above, to increase capture of relevant information, which can help mitigate publication bias. We conducted dual data extraction with cross-check by a third reviewer rather than a single extraction and quality check by a second reviewer to ensure accuracy of quantitative data.

## Results

### Infant exposure to contaminants from HM and/or IF of infants in the United States

#### Search results

A total of 6255 unique records were screened from the database search, and 686 full texts were reviewed. For the SR about infant exposure to contaminants from HM and/or IF, 679 of 686 full texts were excluded because they reported data on HM or IF only and did not report data on infant biospecimen contaminant concentrations. Therefore, the 9 full texts remaining underwent a second round of confirmatory review. Two were excluded due to outcome or population: 1 assessed contaminant in cord blood [49], which was considered ineligible because exposure to HM and/or IF did not precede biospecimen collection, and the other assessed contaminants in naturally exfoliated teeth, which occurs after 12 mo of age [50] (Supplemental Table 8).

Of 544 additional unique records identified via other methods, 57 articles were assessed for eligibility at the full-text level. Two were identified during backward citation searching, and both were excluded: 1 was a duplicate with the database search [51], and 1 was excluded due to population (age ranged from 14 d to 746 d and could not be stratified by those ≤ and >12 mo [52]). Another full text was identified from the manual search of the *Journal of Environmental Exposure Assessment,* but was excluded because only persistent organic pollutants were assessed as an outcome [53], which were not contaminants of interest for our review.

Fifty-four full-text articles were identified from the post hoc search of the HOME study website (*n* = 24), the HOME study clinicaltrials.gov registry (*n* = 22), and the Rochester lead-in-dust study website (*n* = 8). All 54 articles were excluded (detailed exclusion reasons are shown in Supplemental Table 9) due to publication type (*n* = 1), duplicates with the database search (*n* = 6), population (*n* = 35 total; *n* = 10 maternal samples only, *n* = 24 in children >12 mo, and *n* = 1 nonhuman study), no eligible contaminant outcome measured (*n* = 11), and 1 article measured blood lead in infants at 6 mo after a housing renovation intervention, but contaminant concentrations were not reported in relation to infant feeding (HM only, IF only, or HM and IF) [54].

**FIGURE 2:**
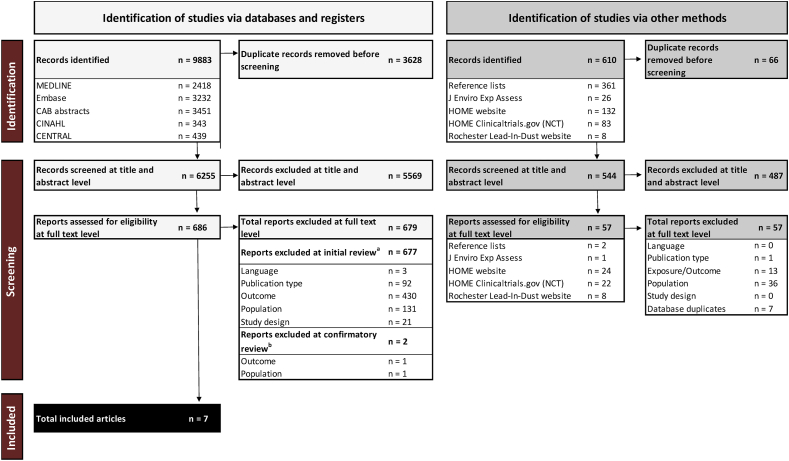
PRISMA chart for “What are biospecimen concentrations of arsenic, cadmium, lead, mercury, or PFAS of infants living in the United States that are consuming human milk and/or infant formula?” (PROSPERO: CRD42024530344). HOME, health outcomes and measures of the environment; J Enviro Exp Assess, Journal of Environmental Exposure Assessment; PFAS, per- and polyfluoroalkyl substances; NCT, national clinical trial. ^a^During initial review, the following data were extracted: the contaminant assessed, whether the contaminant was assessed in human milk, infant formula, and/or infant biospecimens, and the country of data collection. ^b^These data were used to then identify articles that met the inclusion criteria for any of the systematic review questions. Using Stata (StataCorp LLC), the articles were split into datasets specific to each research question, and then the reviewers confirmed whether the inclusion or exclusion criteria were met, specific to each question.

A total of 7 articles were included (Figure 2) that assessed arsenic, cadmium, lead, and/or mercury concentrations in urine and/or blood of infants consuming HM and/or IF aged ≤12 mo living in the United States. No articles were found that assessed PFAS in this population.

#### Study characteristics

**FIGURE 3:**
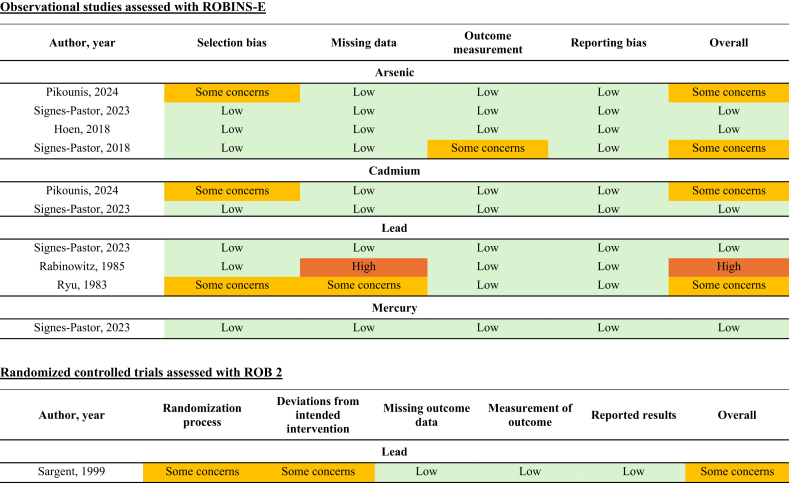
Risk of bias assessment based on study design. See Supplemental Table 7 for operationalization of the ROBINS-E tool specific to this research question. ROBINS-E, risk of bias in nonrandomized studies of exposures. ROB 2, A revised Cochrane risk of bias tool for randomized trials.

Among the 7 included articles, 4 reported data from the New Hampshire birth cohort (NHBC). For this prospective cohort, data were collected between 2009 and 2019. Arsenic (*n* = 4 articles) [51,[55], [56], [57]], cadmium (*n* = 2) [55,56], lead (*n* = 1) [56], and mercury (*n* = 1) [56] were measured via inductively coupled plasma mass spectrometry in feces-free spot urine collected with supplied diapers and cotton pads (Table 1 [51,[55], [56], [57]]). The ROB for the NHBC articles was either low or had some concerns due to selection bias or outcome assessment (Figure 3), and all had acceptable analytic methods.

**TABLE 1:** Summary of urinary metal analyses from infants consuming human milk and/or infant formula in the New Hampshire birth cohort (*n* = 4 articles).

Author, year	Sample characteristics	Total arsenic (μg/L)	Cadmium (μg/L)	Lead (μg/L)	Mercury (μg/L)
Pikounis et al. [55], 2024	Infants with spot urine, 3-d food diary, and relevant covariates at 6 wk old; data collected in 2012‒2019; data reported as geometric mean (95% confidence interval)	All at 6 wk (*n* = 462): 0.23 (0.21‒0.25) Exclusively fed HM (*n* = 338): 0.17 (0.15‒0.18) Exclusively fed formula (*n* = 44): 0.85 (0.66‒1.09) Combination fed (*n* = 80): 0.42 (0.35‒0.5)	All at 6 wk (*n* = 462): 0.14 (0.13‒0.15) Exclusively fed HM (*n* = 338): 0.15 (0.13‒0.16) Exclusively fed formula (*n* = 44): 0.12 (0.09‒0.16) Combination fed (*n* = 80): 0.14 (0.12‒0.16)	—	—
Signes-Pastor et al. [56], 2023	Infants with spot urine and 3-d food diary at 6 wk and 1 y^1^; data collected in 2014‒2019; subset of participants from Pikounis [55], 2024; data reported as median (IQR)	All at 6 wk, exclusively fed HM (*n* = 187): 0.20 (0.06‒0.33) All at 1 y (*n* = 187): 2.31 (1.02‒4.38) Rice consumers at 1 y (*n* = 75): 2.96 (1.63‒6.78) Nonrice consumers at 1 y (*n* = 72): 1.88 (1.06‒3.42)	All at 6 wk, exclusively fed HM (*n* = 187): 0.12 (0.08‒0.20) All at 1 y (*n* = 187): 0.13 (0.08‒0.22) Rice consumers at 1 y (*n* = 75): 0.26 (0.160‒0.38) Nonrice consumers at 1 y (*n* = 72): 0.25 (0.167‒0.33)	All at 6 wk, exclusively fed HM (*n* = 187): 0.55 (0.30‒0.80) All at 1 y (*n* = 187): 0.57 (0.34‒1.17) Rice consumers at 1 y (*n* = 75): 0.84 (0.362‒1.53) Nonrice consumers at 1 y (*n* = 72): 0.60 (0.36‒1.17)	All at 6 wk, exclusively fed HM (*n* = 187):^2^ 0.14 (0.04‒0.23) All at 1 y (*n* = 187): 0.17 (0.08‒0.35) Not assessed for rice consumers vs. nonrice consumers because >60% of values were imputed
Hoen et al. [57], 2018	Infants with urine and stool samples for microbiome analysis and 3-d food diary at 6 wk old; data collected in 2009; data reported as mean with no variability statistic reported	All infants (*n* = 204): 0.6 (range: ≤0.05‒4.8) Exclusively HM fed (*n* = 146): 0.5 Exclusively fed formula (*n* = 9): 1.2 Combination fed (*n* = 49): 0.9	—	—	—
Signes-Pastor et al. [51], 2018	Infants with no introduction to solid foods at 4 mo old; infants weaned with solid foods, in addition to breast milk or formula, at 6 mo; data collected in 2009; data reported as median (range)	Before weaning (4-mo-old) All infants (*n* = 15): 0.265 (0.139‒1.496) Exclusively fed HM (*n* = 11): 0.241 (0.139‒1.326) Exclusively fed formula (*n* = 1): 1.496 (1.496‒1.496) Combination fed (*n* = 3): 1.119 (0.188‒1.266) During weaning (6-mo-old) All infants (*n* = 15): 0.999 (0.170‒11.950) Fed HM, no formula (*n* = 10): 0.552 (0.170‒5.088) Fed formula, no HM (*n* = 3): 1.525 (1.284‒3.271) Combination fed (*n* = 2): 9.456 (6.959‒11.950)	—	—	—

Total arsenic values were reported in each article. Pikounis et al. [55] and Signes-Pastor et al. [51,56], reported inorganic + MMA + DMA minus arsenobetaine, and Hoen et al. [57], reported total arsenic including arsenobetaine. Individual values for inorganic, MMA, DMA, and arsenobetaine were reported by Signes-Pastor et al. [51], and are shown in Supplemental Table 10.

All infants were from the New Hampshire birth cohort. All contaminants were measured via coupled plasma mass spectrometry in feces-free spot urine collected with supplied diapers and cotton pads. These collection and analytic methods were “acceptable” based on the technical expert panel’s critical appraisal, except for the measurement of mercury, in which there were some concerns about the analytic method. This cohort was funded by the National Institute of Environmental Health Sciences, US Environmental Protection Agency, the NIH, and the Neukom Institute at Dartmouth College.

Abbreviations: DMA, dimethylarsinic acid; HM, human milk; IF, infant formula; MMA, monomethylarsonic acid.

1 All infants were exclusively fed HM at 6 wk of age. The proportion consuming HM and/or IF is not reported at 1 y of age.

2 95% of all samples were above machine detection limits, except mercury, in which only 19%‒31% of samples were detected and thus imputed.

The remaining 3 articles reported blood lead concentrations collected from different samples of infants between 1975 and 1994 in 1 randomized controlled trial [58], 1 nonrandomized trial [59], and 1 cross-sectional analysis of a prospective cohort study [60] (Table 2 [58‒60]). Among these 3 studies, samples were collected and analyzed in different ways, as outlined in Table 2. In the 2 trials, formulas were provided to infants (iron-fortified formula with or without calcium glycerophosphate supplementation [58] or a ready-to-feed formula [59]) with repeated blood lead measures over time. Two articles were rated as some concerns for ROB, and 1 was rated high due to selection bias and missing data (Figure 3); all had acceptable analytic methods.

**TABLE 2:** Summary of blood lead analyses from infants consuming human milk and/or infant in the United States (*n* = 3 articles).

Author, year; study design	Data collection	Infant characteristics	Feeding practices	Blood lead (*μ*g/dL)	Sample collection; analytic methods	Funding source
Sargent et al. [58], 1999^1^; randomized controlled trial	Lawrence, Massachusetts, 1991‒1994 (Lawrence women, infants and children program)	Control group: Median age (SD) 3.1 (1.4) mo; mean weight mean ± SD 6.7 (1.2) kg; 92% Latino Supplementation group: Median age (SD) 3.2 (1.3) mo; mean weight (SD) 6.9 (1.2) kg; 92% Latino	Control group: Standard, iron-fortified infant formula (Enfamil, Mead Johnson Co., Evansville, IN) with 465 mg calcium and 317 mg phosphorus per liter Supplementation group: Standard, iron-fortified infant formula (Enfamil, Mead Johnson Co., Evansville, IN) with 1800 mg calcium and 1390 mg phosphorus per liter	Control group median ± SD Baseline (*n* = 52): 2.48 ± 1.24^2^ 4 mo (*n* = 45): 4.55 ± 2.28 9 mo (*n* = 41): 4.97 ± 2.69 No difference over time, *P* > 0.05 Supplementation group Baseline (*n* = 51): 2.48 ± 1.45 4 mo (*n* = 44): 3.52 ± 1.66 9 mo (*n* = 40): 4.76 ± 3.52 No difference over time, *P* > 0.05 No difference between groups at any time point, *P* > 0.05	Antecubital area through a butterfly and syringe; Anodic stripping voltammetry (acceptable)^3^	Bureau of Maternal and Child Health, Health Resources and Services Administration, Department of Health and Human Services
Rabinowitz et al. [60], 1985; prospective cohort study	Boston, Massachusetts, 1979‒1981 (Boston lying-in hospital cohort)	Samples taken at 6 mo; participants selected from the highest, lowest, and centermost deciles of blood lead from the cohort; 87% White	Formulas reported were Similac, Enfamil, and Isomil; the number of those fed HM vs. formula NR	All infants (*n* = 22) mean ± SE: 6.2 ± 0.5 Fed formula (*n* = NR): 5.6 ± 0.5 Fed HM (*n* = NR): 7.6 ± 0.6	Capillary blood, collection methods NR; acid digestion and ESA model 3010 voltameter (acceptable)^3^	National Institute of Child Health and Human Development
Ryu et al. [59], 1983; nonrandomized trial	Iowa City area, Iowa, 1975‒1976	6‒9 d old at enrollment; birth weights were >2450 g; 100% White	Formula-fed infants enrolled and supplied ready-to-feed formula (67 kcal/dL) in glass bottles until 111 d old. Then 4 infants went on to receive commercially available milk-based formula supplied in quart cans until 196 d old, whereas 10 others received homogenized cow milk in cartons from a local dairy farm, and 3 received heat-treated cow milk supplied in cans	Formula-fed mean ± SD 8 d old (*n* = 23): 8.9 ± 3.2 28 d old (*n* = 23): 5.8 ± 2.2 56 d old (*n* = 23): 5.1 ± 1.7 84 d old (*n* = 23): 5.4 ± 2.8 112 d old (*n* = 23): 6.1 ± 1.7 Combined data for those receiving HM or IF cans 140 d old (*n* = 7): 9.3 ± 4 168 d old (*n* = 7): 12.1 ± 4 196 d old (*n* = 7): 14.4 ± 4.4	Collected from an external jugular vein; Flameless atomic absorption spectrophotometry with a graphite furnace (acceptable)^3^	Public Health Service and the Food and Drug Administration

Abbreviations: HM, human milk; IF, infant formula; NR, not reported.

1 The authors state, “Parents were asked to give only the study formula to their infants and not to introduce cow milk until after completion of the study.” Assumingly, this study excludes infants who were HM fed.

2 Converted to μg/dL= μmol/L × 20.7.

3 Based on the technical expert panel’s critical appraisal.

#### Arsenic

Urinary arsenic concentrations were reported in 4 articles [51,[55], [56], [57]] from the NHBC (Table 1). Of these 4, 2 assessed cross-sectional concentrations of urinary arsenic at 6 wk of age [55,57]. One of these articles reported a geometric mean of urinary total arsenic (minus arsenobetaine) as 0.23 μg/L (95% confidence interval (CI): 0.21, 0.25 μg/L) for 462 infants between 2012‒2019 [55]. The other article reported a mean urinary total arsenic concentration (including arsenobetaine) as 0.6 μg/L (range: ≤0.05‒4.8) for 204 infants in 2009 [57]. Possible explanations for differences include using a geometric mean, reduced variability due to the larger sample size in the newer article, inclusion or exclusion of arsenobetaine, or differences in analytic methods.

Urinary arsenic concentrations increased as the infants aged in the NHBC. Specifically, median urinary arsenic concentrations [sum of inorganic, monomethylarsonic acid (MMA), and dimethylarsinic acid (DMA) but not including arsenobetaine] increased 14.8-fold from 6 wk to 1 y for 187 infants in 2014‒2019 (*P* < 0.001) [56]. In a sample of 15 infants in 2009, median urinary concentrations increased by 1.5-fold for inorganic (*P* = 0.04), 5.5-fold for MMA (*P* = 0.002), 5.8-fold for DMA (*P* = 0.01), and 3.8-fold for sum of these 3 (*P* = 0.01) from 4 mo to 6 mo of age (arsenobetaine was not statistically significant, *P* = 0.69; data shown in Supplemental Table 10) [51].

Three articles reported differences in urinary arsenic concentrations associated with HM or IF intake. Urinary arsenic was higher for infants exclusively fed IF, followed by combination-fed (IF and HM) infants, and was lowest among those exclusively fed HM (*P* < 0.0001) for 462 infants in 2012‒2019 [55]. This pattern was also observed in 15 infants in 2009 for inorganic arsenic, MMA, DMA, and total arsenic (sum of inorganic, MMA, and DMA minus arsenobetaine; data shown in Supplemental Table 10) [51].

Urinary arsenic concentrations increased with age, coinciding with the introduction of complementary foods. Infants (*n* = 187 in 2014‒2019) who consumed rice products at 1 y of age had 1.6-fold higher median urinary arsenic concentration compared with nonrice consumers (*P* = 0.001) [56]. An increase in urinary arsenic concentrations was also seen in the smaller subset of 15 infants in 2009 with the introduction of rice cereal (*P* = 0.03), as well as fruits (*P* = 0.03) and vegetables (*P* = 0.01) compared with paired pre-weaning samples [51].

All evidence for arsenic was from a single cohort in 1 geographic location, with little demographic or geographic variability limiting generalizability to the entire US population. Additionally, the 4 articles that assessed arsenic concentrations reported total arsenic, only 1 separately reported inorganic arsenic concentrations [51], which is of particular concern [61]. Therefore, the evidence did not support a conclusion on concentrations of measured arsenic exposure of infants consuming HM and/or IF in the United States.

#### Cadmium

Urinary cadmium concentrations were reported in 2 articles [55,56] from the NHBC (Table 1). Geometric mean urinary cadmium concentration from infants at 6 wk of age (*n* = 462, data collected in 2012‒2019) was 0.14 μg/L (95% CI: 0.13, 0.16 μg/L) [55]. Urinary cadmium concentrations were not significantly different among infants fed HM, IF, or both (*P* = 0.065) [55] or between infants fed rice products compared with infants not fed rice products (*P* = 0.118) [56]. In a subset of this cohort (*n* = 187, in 2014‒2019), paired median cadmium concentrations increased significantly from 0.12 *μ*g/L (IQR = 0.08‒0.20) at 6 wk to 0.13 *μ*g/L (IQR = 0.08‒0.22) at 1 y (*P* = 0.046) [56].

All evidence for cadmium was from a single cohort in 1 geographic location, with little demographic variability limiting generalizability to the entire US population. Therefore, the evidence did not support a conclusion on concentrations of measured cadmium exposure of infants consuming HM and/or IF in the United States.

#### Lead

Lead was assessed in urine in 1 study (NHBC) (Table 1) [56] and in blood from 3 studies [58‒60]. From the NHBC data from 2014‒2019, the median urinary lead concentrations increased from 0.55 μg/L (IQR = 0.30‒0.80) at 6 wk to 0.57 μg/L (IQR = 0.34, 1.17) at 1 y of age (paired samples t test *P* = 0.012) [56]. Lead concentrations did not differ between rice consumers and nonconsumers (*P* = 0.187) at 1 y of age [56]. The relationship to feeding practice (e.g., HM, IF, or both) was not assessed.

Blood lead concentrations were collected between 1975 and 1994 (Table 2). Concentrations across the 3 studies ranged from 2.48 ± 1.24 μg/dL at 3 mo [58] to 14.4 ± 4.4 μg/dL at ∼6.5 mo [59]. In Iowa, a nonrandomized intervention with IF showed a change in blood lead concentrations in infants over time. Specifically, concentrations decreased from ∼8 d to ∼2 mo of age (8.9 ± 3.2 μg/dL to 5.1 ± 1.7 μg/dL; *P* < 0.05) but did not change from ∼2 mo to ∼3.5 mo of age [59]. In a prospective cohort study in Boston, blood lead concentrations did not differ between infants fed HM (7.6 ± 0.6 μg/dL) or IF (5.6 ± 0.5 μg/dL) at 6 mo [60]. In a randomized intervention in Massachusetts, blood lead concentrations did not differ from ∼3 mo to 9 mo or between groups fed iron-fortified IF with or without calcium glycerophosphate supplementation [58].

In total, 4 articles reported urinary [56] and blood [[58], [59], [60]] lead concentrations in infants. The results for urinary lead were more recent (2014‒2019) but came from a single cohort in 1 geographic location with little demographic variability, and the results for blood lead were >30 y old. Collectively, these limitations indicate a lack of generalizability to the current US infant population. Therefore, the evidence did not support a conclusion on concentrations of measured lead exposure in biospecimens of infants consuming HM and/or IF in the United States.

#### Mercury

Urinary total mercury concentrations were reported in 1 article [56] from the NHBC with samples collected in 2014‒2019 (Table 1). Median mercury concentrations were 0.14 μg/L (IQR = 0.04‒0.23) in infants exclusively fed HM at 6 wk and 0.17 μg/L (IQR = 0.08‒0.35) in the same infants consuming rice/rice products at 1 y of age, demonstrating an increase over time (paired samples t test *P* = 0.007). Differences based on consumption of HM and/or IF or other dietary exposures were not assessed at the 1-y timepoint. Additionally, methylmercury was not reported at either timepoint.

Evidence from 1 article about a single cohort in 1 geographic location with little demographic variability did not support a conclusion on mercury exposure of infants consuming HM and/or IF in the United States.

### Contaminant bioavailability from HM or IF

#### Search results

**FIGURE 4:**
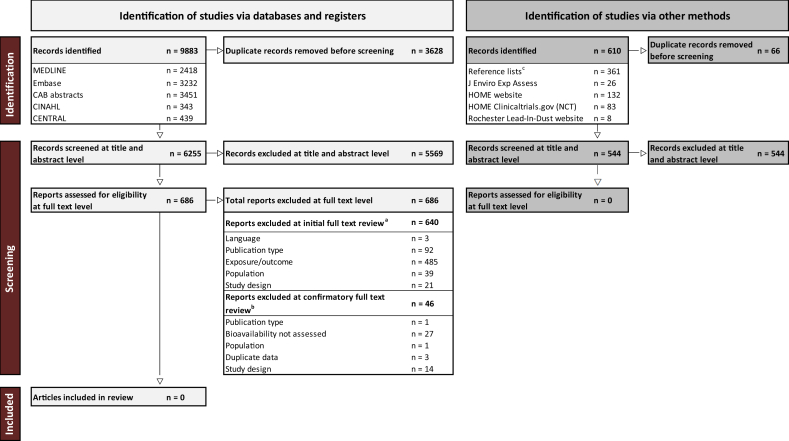
PRISMA chart for research questions related to bioavailability (PROSPERO: CRD42024530332, CRD42024530336, and CRD42024530339). ^a^During initial review, the following data were extracted: the contaminant assessed, whether the contaminant was assessed in human milk, infant formula, and/or infant biospecimens, and the country of data collection. ^b^This data was used to then identify articles that met the inclusion criteria for any of the systematic review questions. Using Stata (StataCorp LLC), the articles were split into datasets specific to each research question, and then the reviewers confirmed whether the inclusion or exclusion criteria were met, specific to each question. There were no articles that met the inclusion criteria for the first question about bioavailability (question #4 in Supplemental Table 1), which was a requirement to meet the inclusion criteria for subsequent bioavailability SRs (questions 5 and 6 in Supplemental Table 1). Therefore, this PRISMA diagram is representative of all 3 SRs related to bioavailability. ^c^Corresponds to articles identified from the backward citation of the question related to infant exposure to contaminants, as these articles were screened for all questions. SR, systematic review; NCT, national clinical trial.

For the 3 SRs related to bioavailability, 686 full texts identified from the database search were reviewed, and 640 were excluded due to lack of relevancy to the SRs’ questions. The 46 relevant full texts from the database search underwent a second round of confirmatory review, but none met the inclusion criteria, mostly because bioavailability was not assessed (exclusion reasons are shown in Supplemental Table 8). Of 544 additional unique records identified via other methods, no articles were included at the title/abstract level and therefore were not assessed for eligibility at the full-text level. Thus, no studies were identified on the bioavailability of arsenic, cadmium, lead, mercury, or PFAS in HM or IF (Figure 4).

#### Post hoc assessment of associations

During screening, articles were tagged if they did not report bioavailability but did meet other inclusion criteria and reported associations between contaminant concentrations in HM or IF and contaminant concentrations in infant biospecimens. Nine articles were identified that included contaminant concentrations in HM and infant biospecimens (no articles were identified that included contaminant concentrations in IF and infant biospecimens).

These studies reported statistically significant correlations between contaminant concentrations (arsenic [62], mercury [63,64], lead [60], PFOA and PFNA[65]) in HM consumed by infants and contaminant concentrations in infant urine or whole blood, but not red blood cells [66] or stool [63,[67], [68], [69]] (Table 3 [60,[62], [63], [64], [65], [66], [67], [68], [69]]). Associations between contaminants in HM and infant hair samples for 2 [63,67] of 3 [68] studies that assessed mercury were not statistically significant for total or methylmercury. Conclusions about bioavailability of these contaminants from HM cannot be made from these studies. However, this information may be useful to inform future research priorities on the topic.

**TABLE 3:** Articles assessing associations between contaminant concentrations in human milk and/or infant formula and contaminant concentrations in infant biospecimens.

Author, year	Country	Infant age, feeding mode	Results
**Arsenic**			
Linares et al. [62], 2024	Peru	Mean age = 11.2 (range: 3‒20) mo; all fed human milk; 43% also had formula at ≤6 mo old and 52% also consumed milk (mainly evaporated milk) at ≥7mo	A linear regression model suggested that every 1 μg/L increase in HM arsenic concentration was associated with a 1.40 ± 1.08 (SE) μg/dL increase in infant blood arsenic concentration (*P* < 0.001, *n* = 40)
Astolfi et al. [69], 2019	Italy	Probiotic supplement/placebo was given for the last 4 weeks of gestation and during the first month of lactation, but the infant’s age was not specified otherwise; exclusively fed HM during the study	Not analyzed because levels were below LOD. C: 13 mothers, 13 infants T: 16 mothers, 18 infants^1^
Sakamoto et al. [66], 2012	Japan	3 mo old; 15 infants were reared on HM, 1 was reared mainly on HM and additional milk formula beginning at 5 wk	No significant correlations (Spearman rank coefficient) between As in HM and As in infant red blood cells (data not reported, 16 infant/mother dyads, but there are only 9 HM samples described)
**Cadmium**			
Astolfi et al. [69], 2019	Italy	Probiotic supplement/placebo was given for the last 4 wk of gestation and during the first month of lactation, but the infant’s age was not specified otherwise; exclusively fed HM during the study	Kendall’s tau-b correlation for HM sampled at all stages of lactation. C: 13 mothers, 13 infants T: 16 mothers, 18 infants^1^ C: Cd in infant stools and Cd in HM K = ‒0.153, *P* = 0.112 T: Cd in infant stools and Cd in HM K = ‒0.138, *P* = 0.277 C: Cd in infant stools and Hg in HM K = ‒0.022, *P* = 0.819 T: Cd in infant stools and Hg in HM K = 0.063, *P* = 0.622
Sakamoto et al. [66], 2012	Japan	3 mo old; 15 infants were reared on HM, 1 was reared mainly on HM and additional milk formula beginning at 5 wk	No significant correlations (Spearman rank coefficient) between Cd in HM and Cd in infant red blood cells (data not reported, 16 infant/mother dyads but there are only 9 HM samples described)
**Mercury**			
Astolfi [69], 2019	Italy	Probiotic supplement/placebo was given for the last 4 wk of gestation and during the first month of lactation, but the infant’s age was not specified otherwise; exclusively fed HM during the study	Kendall’s tau-b correlation for HM sampled at all stages of lactation C: 13 mothers, 13 infants T: 16 mothers, 18 infants^1^ C: Hg in infant stools and Cd in HM K = 0.067, *P* = 0.851 T: Hg in infant stools and Cd in HM K = ‒0.143, *P* = 0.621 C: Hg in infant stools and Hg in HM K = 0.200, *P* = 0.573 T: Hg in infant stools and Hg in HM K = ‒0.074, *P* = 0.802
Al-Saleh et al. [63], 2016	Saudi Arabia	3‒12 mo old; 40.1% were breastfed when enrolled, and 59.9% were breastfed with a median duration of 2 mo, but not currently breastfeeding	Pearson correlations (imputed values for less than minimum detection limit as 1/2 × minimum detection limit) Total Hg infant urine and HM 0.149 *P* = 0.028, *n* = 217 Total Hg infant hair and HM 0.063 *P* = 0.283, *n* = 288 MeHg infant hair and HM ‒0.022 *P* = 0.727, *n* = 245
Okati et al. [67], 2013	Iran	<6 mo old; fed HM only	Pearson correlation between mercury in HM and mercury in infant hair: *r* = 0.02, *P* = 0.06 (*n* = 82)
Sakamoto et al. [66], 2012	Japan	3 mo old; 15 infants were reared on HM, 1 was reared mainly on HM and additional milk formula beginning at 5 wk of age	No significant correlations (Spearman rank coefficient) between Hg in HM and Hg in infant red blood cells (data not reported, 16 infant/mother dyads but there are only 9 HM samples described)
Bjornberg et al. [64], 2005	Sweden	Enrolled at delivery and followed until 13 wk old; 6 infants fed formula and HM, and 14 assumingly exclusively fed HM at 13 wk	Total mercury in HM was not significantly correlated with infant blood inorganic mercury at 13 wk (*r* = 0.17, *P* = 0.50, *n* = 19) Total mercury in HM was correlated with infant blood methylmercury at 13 wk (*r* = 0.55, *P* = 0.01, *n* = 19)
Tatsuta et al. [68], 2024	Japan	32 samples collected from infants aged 0‒4 mo that were consuming infant formula measured by the duplicate diet method	MeHg in formula milk was negatively correlated with total mercury in hair of infants (*r* = ‒0.403, *P* = 0.030)
**Lead**			
Linares et al. [62], 2024	Peru	Mean age = 11.2 (range: 3‒20) mo; all fed HM; 43% also had formula at ≤6 mo old and 52% also consumed milk (mainly evaporated milk) at ≥7 mo	A multiple linear regression model suggested that HM lead concentration was not associated with infant blood lead concentrations after adjusting for the infant’s age (β = 0.11 ± 0.14 μg/dL, *P* = 0.441, *n* = 40)
Astolfi et al. [69], 2019	Italy	Probiotic supplement/placebo was given for the last 4 wk of gestation and during the first month of lactation, but the infant’s age was not specified otherwise; exclusively breastfed during the study period	Not analyzed because levels were below LOD. C: 13 mothers, 13 infants T: 16 mothers, 18 infants^1^
Sakamoto et al. [66], 2012	Japan	3 mo old; 15 infants were reared on HM, 1 was reared mainly on HM and additional milk formula beginning at 5 wk of age	No significant correlations (Spearman rank coefficient) between Pb in HM and Pb in infant red blood cells (data not reported, 16 infant/mother dyads but there are only 9 HM samples described)
Rabinowitz et al. [60], 1985	United States	Infants were enrolled at birth and followed through 6 mo; some infants were breastfed while others were given formula (sample sizes NR)	Spearman rank correlation for infant blood lead concentrations and breastmilk *r* = 0.42, *P* = 0.0003 (*n* = 69)
**PFAS**			
Yao et al. [65], 2023	China	Infants were enrolled at birth and gave samples within the first postnatal week; all infants breastfed (exclusivity or formula use NR)	Correlation of log_10_-transformed for paired samples (*n* = 80) PFOA in HM and infant urine *R*^2^ = 0.29, *P* < 0.01 PFOS in HM and infant urine *R*^2^ = 0.01, *P* = 0.15 PFNA in HM and infant urine *R*^2^ = 0.09, *P* < 0.01 PFHxS in HM and infant urine NR

Abbreviations: As, arsenic; C, control; Cd, cadmium; Hg, mercury; HM, human milk; LOD, limit of detection; MeHg, methylmercury; NR, not reported; Pb, lead; PFHxS, perfluorohexanesulfonic acid; PFNA, perfluorononanoic acid; PFOA, perfluorooctanoic acid; PFOS, perfluorooctanesulfonic acid; T, treated.

1 These are numbers reported in the paper, however the number of mothers and infants do not match and twins were excluded from the original study. Therefore, this may be a typo in the article.

## Discussion

Our objective was to assess the bioavailability of and US infant exposure to arsenic, cadmium, lead, mercury, and legacy PFAS in HM and IF. We found a few studies that assessed arsenic, cadmium, lead, mercury, or PFAS exposure as measured in biospecimens from infants consuming HM and/or IF in the United States. The available evidence for arsenic, cadmium, lead, and mercury was from a single cohort in New Hampshire or was collected >30 y ago, limiting generalizability to the diverse group of infants in the United States currently. Additionally, no articles were identified that assessed contaminant bioavailability from HM or IF, which is essential information for calculating exposure estimates.

Most studies identified in our SR were conducted in the Northeast, thus the results are not generalizable to infants across the United States. Geographic location can affect contaminant exposure, such as proximity to industrial areas, mines, landfills, and waste discharge points [70] and natural sources of contaminants [71]. For example, infants in the NHBC (the population in 4/7 included articles) lived in rural New Hampshire, where contaminant exposure from air pollution is low [72] yet arsenic exposure from the granite bedrock is high [73,74]. The New Hampshire Department of Environmental Services reports ∼25% probability of naturally occurring arsenic in private wells [75] and adopted a maximum contaminant level of 5 parts per billion for arsenic in public water systems in 2021 [76]. Water contamination like this can increase exposure of pregnant and lactating individuals and potential contaminant transfer into HM [14] as well as introduce contaminants into IF during preparation. Future research could include other regional cohorts like the NHBC to monitor contaminant exposure in different communities, prioritizing high-risk geographic areas of the United States. Supplementing US data with data from countries that have similar environmental conditions may be necessary if new data collection is not possible.

Beyond limited geographic representation, the evidence base also lacked demographic and socioeconomic diversity needed to understand infant exposure risk on a national level. Most infants studied were described as White with high socioeconomic position, which are characteristics associated with lower contaminant exposure [77,78]. For example, data from the NHANES suggest that lead exposure is twice as high between lower-socioeconomic groups [79], and individuals identifying as Black, Hispanic, or multiracial have higher exposures to arsenic, cadmium, lead, and mercury than non-Hispanic White individuals, including pregnant women [78,80,81]. These disparities highlight the need to consider demographic and socioeconomic heterogeneity in estimates of infant exposure to contaminants from HM across the country.

Existing US surveillance systems, such as NHANES, could be leveraged to assess infant contaminant exposure using biospecimens. Currently in NHANES, biospecimens are not collected from individuals aged <1 y, likely due to funding, challenges in obtaining parental consent, and difficulty in infant sample collection. Advancements in recruitment efforts would be needed, given that the 2021‒2023 NHANES cycle included only 133 infants for the medical examination [82] with a response rate of <25% [82]. Small sample sizes and low response rates increase risk of selection bias and compromise statistically reliable and nationally representative estimates. Significant investments would be needed [83] to incorporate infant biomonitoring and estimate corresponding consumption of HM and IF. This type of research would be critical for establishing contaminant reference values that would allow for assessing trends over time and provide a comparison for healthcare providers to better monitor their patients’ exposure.

The results of our SRs are bound by the real-world challenges of collecting biospecimens, particularly blood, from infants. Researchers often must sacrifice scientific rigor for ethical and practical concerns for such a vulnerable target population. For example, we intended to assess exposure to total and inorganic arsenic, total mercury and methylmercury, and PFAS. However, we did not identify any data on methylmercury or PFAS, likely due in part to the lack of blood collection that is recommended for certain analytes [84,85]. Four of 7 articles [51,[55], [56], [57]] in our SR assessed contaminants in urine, which is easier to collect, but it is often inferior to blood for some analytes. Future research can continue to collect spot and 24-h urine using newborn U-bags [86] for cadmium, total arsenic, and elemental and inorganic mercury [84]. However, blood would be needed to analyze various contaminant forms (e.g., organic and inorganic arsenic) and species (e.g., the organic species of MMA and DMA or inorganic species of arsenite and arsenate) that pose a higher risk for infants.

Standard analytical methods are established for heavy metals but are still evolving for PFAS, requiring standardization for high-quality large-scale biomonitoring. Preparation, storage, and processing of HM and IF samples for PFAS analysis are challenging due to potential introduction of extrinsic PFAS, given their ubiquity in consumer goods [87,88]. Future research efforts assessing infant PFAS exposure need to carefully consider challenges at each stage of data collection, including blood collection (i.e., prioritizing serum), preparation, storage, instrument selection, quality assurance and quality control, instrument calibration, and transparency in reporting of methods and quality assurance data [88]. These considerations will help ensure accurate and reliable PFAS exposure prevalence estimates to inform downstream risk analyses.

Urinary arsenic was reported in all articles using data from the NHBC with assessments spanning from 6 wk to 1 y of age. Articles reporting longitudinal data consistently found an increase in arsenic concentrations with age, coinciding with the introduction of complementary foods. A possible explanation for higher urinary concentrations in older infants may be increased consumption of folic acid fortified foods (e.g., rice cereal), which promote arsenic methylation and urinary excretion [27,28]. By 12 mo of age, infant renal function (i.e., glomerular filtration rates and tubular function) more closely resembles that of adults [89], potentially contributing to higher concentrations relative to younger infants. Thus, the increase in urinary arsenic with age may not reflect increased exposure but rather improved clearing and decreased circulating levels. These findings underscore the need for additional research to analyze contaminants in both the foods being consumed and coinciding infant biospecimens over biologically relevant lengths of time.

No articles were identified to address the SR questions on bioavailability. This is likely because bioavailability experiments are rare for infant populations. These experiments are burdensome, invasive, could involve ingestion of radioactive or stable isotopes, and require many repeated measures of various biospecimen types over time, along with well-documented dietary intake, in addition to the ethical considerations [90]. Therefore, observational evidence will likely remain the highest quality evidence available to address this topic. Observational data, including the 9 articles that we summarized post hoc, may offer useful insight for understanding the bioavailability of contaminants from HM and IF if the results can be corroborated by mechanistic study designs, such as animal or *in vitro* digestion models. Future SRs are needed to synthesize findings from such mechanistic study designs and consider baseline contaminant exposure as a potential moderator that may affect bioavailability.

Our SR followed rigorous methodological standards. First, the protocol was developed with input from a TEP, registered a priori, followed reporting guidance from PRISMA [31], and met the AMSTAR 2 criteria for a high-quality review [32]. Second, a comprehensive search strategy was developed by SR information specialists, peer-reviewed by a third librarian, and included 5 databases. Gray literature, clinical trial registries, and non-English articles were excluded, which may increase the risk for publication bias. However, the objective of this research was to specifically summarize peer-reviewed scientific evidence only.

A limitation of our SR is that we could not assess exposure to contaminants from HM or IF directly because biospecimen concentrations represent exposure from all sources, and not just foods. To ensure that HM and IF were at least possible exposure sources, we restricted to articles that reported contaminant concentrations of infant biospecimens in relation to the consumption of HM and/or IF (i.e., contaminant concentrations needed to be reported based on infant feeding of HM only, IF only, or HM and IF). Additionally, we excluded biospecimen types, such as cord blood and meconium, which were collected prior to the infant’s first exposure to HM and/or IF. Future research is needed to assess contaminant concentrations in infant biospecimens alongside samples of consumed HM and/or IF to more directly inform exposure from these foods.

### Future research summary

Our findings illuminate critical research gaps and opportunities for future research to estimate infant exposure to potential contaminants in HM and/or IF. First, national biomonitoring in the United States could estimate infant contaminant exposure from all sources, accounting for factors such as location, demographics, and socioeconomic status. These existing surveillance systems, as well as research partnerships with pediatric practices and local health departments (standard well-child visits are at 3‒5 d, 1 mo, 2 mo, 4 mo, 6 mo, 9 mo, and 12mo) [91] could also facilitate sample collection of HM and IF that the infant consumes to estimate bioavailability ratios. Inclusion of mother-infant pairs, maternal dietary data, and HM or IF samples could inform how contaminants transfer from maternal food exposure to maternal tissues and HM, and finally to the infant. Additionally, SRs are needed that synthesize findings from animal or *in vitro* digestion models that indirectly assess bioavailability of contaminants from HM or IF. Collectively, these efforts will strengthen the evidence base for investigating associations between contaminant exposure from foods and infant health and development. These research efforts would inform policymakers as they prioritize public health efforts (e.g., reduce maternal contaminant exposures during pregnancy and lactation [92,93] and increase regulatory testing of IF [2]) to help monitor and mitigate infant exposure to contaminants.

In conclusion, there was a lack of contemporary data from geographically and demographically diverse populations across the United States to understand infant exposure to arsenic, cadmium, lead, mercury, and PFAS from HM and/or IF in the United States. Additionally, no research assessed contaminant bioavailability in HM or IF or factors that may affect the bioavailability of contaminants or essential nutrients. Notably, this lack of evidence does not indicate a lack of exposure for infants. Rather, these findings underscore the necessity for extensive biomonitoring to understand infant exposure to these contaminants to better inform policymakers in efforts to reduce contaminant exposures from food.

## Author contributions

The authors’ responsibilities were as follows – MKS, AJM: designed the research; KGD, MMF: served on the technical expert panel (TEP) to inform the protocol; ALG, CAH: served on the TEP to critically appraise sample collection procedures and contaminant analytic methods; MJF, KMH: designed and conducted the literature search; LEO, RCT, CNL, AAB, SMA, TB, AJM, MKS: conducted the research; LEO, RCT, CNL, AAB: prepared the data; LEO: synthesized the data; LEO, RCT, CNL: wrote the paper; AAB, SMA, TB, MJF, KMH, KGD, MMF, ALG, CAH, AJM, MKS: reviewed and edited the paper; and all authors: read and approved the final manuscript.

## Funding

This project was supported by the United States Department of Health and Human Services – Food and Drug Administration. The sponsor provided the scientific questions and scope and agreed to question refinements. Evidence Center analysts were responsible for developing and registering the protocol and conducting the review.

## Declaration of generative AI and AI-assisted technologies in the writing process

The authors declare that no generative AI or AI-assisted technologies were used in the writing of this manuscript.

## Conflict of interest

The authors report no conflicts of interest.
