# Supplementary material for: Bioavailability of and US Infant Exposure to Arsenic, Cadmium, Lead, Mercury, and Per- and Polyfluoroalkyls from Human Milk and Infant Formula: Results from a Series of Systematic Reviews
Source: Adv Nutr. 2026 Apr 9;17(5):100628. doi: 10.1016/j.advnut.2026.100628 (PMC13156631; doi:10.1016/j.advnut.2026.100628)
Supplement: Multimedia component 1 [file mmc1.docx]

**Bioavailability of and US Infant Exposure to Arsenic, Cadmium, Lead, Mercury, and Per- and Polyfluoroalkyls from Human Milk and Infant Formula: Results from a Series of Systematic Reviews** (O’Connor, et al.)

Supplemental Materials

Contents

[**Supplemental Table 1.** Series of systematic review questions related to potential contaminants in human milk and infant formula. 2](#_Toc228346463)

[**Supplemental Table 2.** Electronic database search strategy for questions 2-6 from Supplemental Table 1. 3](#_Toc228346464)

[**Supplemental Table 3.** Inclusion and exclusion criteria for ‘What are biospecimen concentrations of arsenic, cadmium, lead, mercury, or PFAS of infants living in the United States that are consuming human milk and/or infant formula?’ (PROSPERO: CRD42024530344). 16](#_Toc228346465)

[**Supplemental Table 4.** Inclusion and exclusion criteria for ‘What is the bioavailability of arsenic, cadmium, lead, mercury, or PFAS from human milk and infant formula when consumed by the infant?’ (PROSPERO: CRD42024530332). 18](#_Toc228346466)

[**Supplemental Table 5.** Inclusion and exclusion criteria for ‘What factors impact the bioavailability of arsenic, cadmium, lead, mercury, or PFAS from human milk and infant formula when consumed by the infant?’ (PROSPERO: CRD42024530336). 20](#_Toc228346467)

[**Supplemental Table 6.** Inclusion and exclusion criteria for ‘What is the relationship between arsenic, cadmium, lead, mercury, or PFAS and the bioavailability of other components in human milk and infant formula?’ (PROSPERO: CRD42024530339). 22](#_Toc228346468)

[**Supplemental Table 7.** Operationalization of ROBINS-E^a^ for ‘What are biospecimen concentrations of arsenic, cadmium, lead, mercury, or PFAS of infants living in the United States that are consuming human milk and/or infant formula?’ (PROSPERO: CRD42024530344). 24](#_Toc228346469)

[**Supplemental Table 8.** Excluded full text articles identified from the database search, backwards citation search, or manual search of the *Jornal of Environmental Exposure Assessment* with reasons for exclusion. 25](#_Toc228346470)

[**Supplemental Table 9**: Excluded full text articles identified from the HOME Study website, HOME study trial registry (NCT), or Rochester Lead-in-Dust study website* with reasons for exclusion. 30](#_Toc228346471)

[**Supplemental Table 10**: Measured median (min-max) urinary arsenic species reported in infants by feeding practice (Signes-Pastor, 2018) 36](#_Toc228346472)

[**Supplemental Figure 1.** Analytic framework for ‘What are biospecimen concentrations of arsenic, cadmium, lead, mercury, or PFAS of infants living in the United States that are consuming human milk and/or infant formula?’ (PROSPERO: CRD42024530344). 37](#_Toc228346473)

[**Supplemental Figure 2.** Analytic framework for ‘What is the bioavailability of arsenic, cadmium, lead, mercury, or PFAS from human milk and infant formula when consumed by the infant?’ (PROSPERO: CRD42024530332). 38](#_Toc228346474)

[**Supplemental Figure 3.** Analytic framework for ‘What factors impact the bioavailability of arsenic, cadmium, lead, mercury, or PFAS from human milk and infant formula when consumed by the infant?’ (PROSPERO: CRD42024530336). 39](#_Toc228346475)

[**Supplemental Figure 4.** Analytic framework for ‘What is the relationship between arsenic, cadmium, lead, mercury, or PFAS and the bioavailability of other components in human milk and infant formula?’ (PROSPERO: CRD42024530339). 40](#_Toc228346476)

[**Appendix A.** PRISMA 2020 checklist. 41](#_Toc228346477)

[**Appendix B.** AMSTAR 2 checklist: assessing the methodological quality of systematic reviews. 45](#_Toc228346478)

## **Supplemental Table 1.** Series of systematic review questions related to potential contaminants in human milk and infant formula.

**Systematic review questions:**

1. What is the relationship between exposure to contaminants during pregnancy and lactation and contaminant concentrations in human milk? **(PROSPERO: CRD42024530326)**

- **Publication:** [O'Connor LE, Uffelman CN, Thoerig RC, et al. Arsenic, cadmium, lead, mercury, and PFAS exposure during pregnancy or lactation and respective concentrations in human milk: Systematic review. Environ Res. 2026;290:123433. doi:10.1016/j.envres.2025.123433](https://www.sciencedirect.com/science/article/pii/S0013935125026866?via%3Dihub#sec4)

1. What is the composition/variability of contaminants in human milk and infant formula? **(PROSPERO: CRD42024528756)**

- **Publication:** [Thoerig RC, O'Connor LE, Spill MK, et al. Assessment of arsenic, cadmium, lead, mercury, and per- and polyfluoroalkyl substances concentrations in human milk and infant formula in the United States: a systematic review. Am J Clin Nutr. 2025;122(4):1006-1026. doi:10.1016/j.ajcnut.2025.07.039](https://www.sciencedirect.com/science/article/pii/S0002916525004563?via%3Dihub)

1. What are biospecimen concentrations of arsenic, cadmium, lead, mercury, or PFAS of infants living in the United States that are consuming human milk and/or infant formula **(PROSPERO: CRD42024530344)**
2. What is the bioavailability of arsenic, cadmium, lead, mercury, or PFAS from human milk and/or infant formula when consumed by the infant? **(PROSPERO: CRD42024530332)**
3. What factors impact the bioavailability of arsenic, cadmium, lead, mercury, or PFSA from human milk and/or infant formula when consumed by the infant? **(PROSPERO: CRD42024530336)**
4. What is the relationship between arsenic, cadmium, lead, mercury, or PFAS and the bioavailability of other components in human milk and/or infant formula? **(PROSPERO: CRD42024530339)**

Questions 3-6 are addressed in O’Connor et al., *Bioavailability of and US Infant Exposure to Arsenic, Cadmium, Lead, Mercury, and Per- and Polyfluoroalkyls from Human Milk and Infant Formula: Results from a Series of Systematic Reviews.*

## **Supplemental Table 2.** Electronic database search strategy for questions 2-6 from Supplemental Table 1.

Librarian(s): Margaret Foster, Kyle Holland

Peer-reviewer name and/or organization: Sheila Green, Texas A&M University Medical Sciences Library

Peer-review date: April 2, 2025

There are 2 main concepts required for retrieval in the search grouped as follows:

*Concept 1*: milk consumed by infants (infant formula or human milk) or lactation

*Concept 2*: contaminants (arsenic, cadmium, lead, mercury, or PFAS)

Each concept was built by combining synonyms and relevant thesaurus terms from each database. The PFAS concept built with terms found in another systematic review (Frigerio, Ferrari, and Fustinoni, 2023). The search started with Medline (Ovid) and then translated for the other 4 databases: Embase (Ovid), CINAHL (Ebsco), CAB Abstracts (Ovid), and CENTRAL (Cochrane Library).

Total number of records identified from each database:

**MEDLINE:** 2418

**Embase:** 3232

**CAB Abstracts:** 3451

**CINAHL:** 343

**CENTRAL:** 439

Number of duplicates removed outside of Distiller: 3321

Number of duplicates removed in Distiller: 307

**Manual search of *Journal of Environmental Exposure Assessment***: 26

Number of duplicates removed from journal manual search: 13

Search strategy for each database:

Resources that contributed important and/or substantial elements to the below search strings.

| Resource | Contribution |
| --- | --- |
| Frigerio, G., Ferrari, C.M. & Fustinoni, S. Prenatal and childhood exposure to per-/polyfluoroalkyl substances (PFASs) and its associations with childhood overweight and/or obesity: a systematic review with meta-analyses. *Environ Health* 22, 56 (2023). https://doi.org/10.1186/s12940-023-01006-6 | Many PFAS terms sourced. |

MEDLINE

Database: MEDLINE

Platform: Ovid

Date of search: April 2, 2025

Limits: Language: English; Not animal studies

| Concept | Line | Search Strategy |
| --- | --- | --- |
| **human milk** | 1 | exp Milk, Human/ |
|  | 2 | (((human? or breast* or maternal or mother*) adj milk*) or breastmilk? or colostrum).ti,ab,kf. |
|  | 3 | or/1-2 |
| **infant formula** | 4 | exp Infant Formula/ or exp Bottle Feeding/ |
|  | 5 | (((baby or babies or newborn* or "new born*" or infant* or neonat* or "neo nat*") adj formula*) or bottlefe* or (bottle adj (feed* or fed))).ti,ab,kf. |
|  | 6 | or/4-5 |
| **lactation** | 7 | exp Lactation/ or exp Breast Feeding/ |
|  | 8 | (lactat* or breastfe* or (breast adj (fed or feed*))).ti,ab,kf. |
|  | 9 | or/7-8 |
| **contaminants** | 10 | exp Mercury/ or exp Mercury Compounds/ or exp Organomercury Compounds/ or exp Mercury Poisoning/ |
|  | 11 | (mercur* or methylmercur* or organomercur* or MeHg or CH3Hg or (Hg and (metal* or organometal* or cation* or ion* or isotop* or radioisotop* or element* or microelement* or organic or inorganic))).ti,ab,kf. |
|  | 12 | exp Lead Poisoning/ or exp Lead/ or exp Lead Radioisotopes/ or exp Tetraethyl Lead/ |
|  | 13 | lead.ti,kf,nm. or (pb or 208Pb or organolead* or plumbum or plumbic* or plumbate* or plumbous or tetraethyllead or (lead adj8 ("208" or metal* or organometal* or cation* or ion* or isotop* or radioisotop* or element* or microelement* or organic or inorganic or ore or blood* or chemical? or poison* or contam*))).ti,ab,kf. |
|  | 14 | exp Arsenic Poisoning/ or exp Arsenic/ or exp Arsenicals/ |
|  | 15 | ((arsen$2 not arsenal) or arsen##$3 or arsine$1 or organoars*).ti,ab,kf. |
|  | 16 | exp Cadmium/ or exp Cadmium Poisoning/ or exp Cadmium Radioisotopes/ or exp Cadmium Compounds/ |
|  | 17 | (cadmi* or organocadmi* or (Cd and (metal* or organometal* or cation* or ion* or isotop* or radioisotop* or element* or microelement* or organic or inorganic))).ti,ab,kf. |
|  | 18 | exp Fluorocarbons/ |
|  | 19 | (perfluor* or polyfluor* or methylperfluor* or ethylperfluor* or fluorocarb* or fluorochemical* or fluorosurfactant* or fluorotelomer* or fluorinate* or PFAS$1 or PFBA$1 or PFBS$1 or PFC$1 or PFCA$1 or PFDA$1 or PFDoDA$1 or PFDS$1 or PFECHS$1 or PFESA$1 or PFEtCHxS$1 or PFHp$1 or PFHpA$1 or PFHpS$1 or PFHxA$1 or PFHxS$1 or PFNA$1 or PFNS$1 or PFOA$1 or PFOS$1 or PFOSA$1 or PFPA$1 or PFPeA$1 or PFPeS$1 or PFPiA$1 or PFSA$1 or PFTE$1 or PFTeDA$1 or PFTrDA$1 or PFUnDA$1 or FOSA$1 or EtFOSA$1 or MeFOSA$1 or DONA$1 or FTOH$1 or FTSA$1 or HFPO$1 or LCPFA$1 or SCPFA$1 or Teflon or freon or GenX).ti,ab,kf. |
|  | 20 | Metals, Heavy/ or Heavy Metal Poisoning/ or (heavy adj metal*).ti,ab,kf. |
|  | 21 | or/10-20 |
| ***no animal studies*** | 22 | (exp Animals/ not (exp Animals/ and Humans/)) or exp Animal Experimentation/ or exp Models, Animal/ |
|  | 23 | ((animal? adj2 (study or model* or experiment*)) or mouse or mice or rat or rats or monkey or monkeys or "preclinical study").ti. |
|  | 24 | or/22-23 |
| **Protocol Question 2-6** | 25 | ((3 or 6 or 9) and 21) not 24 |
|  | 26 | ..l/ 25 en=y |

Embase

Database: Embase

Platform: Ovid

Date of search: April 2, 2025

Limits: English Language; Not animal studies

| Concept | Line | Search Strategy |
| --- | --- | --- |
| **human milk & infant formula** | 1 | exp infant feeding/ or exp baby food/ |
|  | 2 | (((human? or breast* or maternal or mother*) adj milk*) or breastmilk? or colostrum or (((baby or babies or newborn* or "new born*" or infant* or neonat* or "neo nat*") adj formula*) or bottlefe* or (bottle adj (feed* or fed)))).ti,ab,kf. |
|  | 3 | or/1-2 |
| **lactation** | 4 | lactation/ or exp breast feeding/ |
|  | 5 | (lactat* or breastfe* or (breast adj (fed or feed*))).ti,ab,kf. |
|  | 6 | or/4-5 |
| **contaminants** | 7 | mercury/ or mercury derivative/ or exp mercurialism/ or exp organomercury compound/ |
|  | 8 | (mercur* or methylmercur* or organomercur* or MeHg or CH3Hg or (Hg and (metal* or organometal* or cation* or ion* or isotop* or radioisotop* or element* or microelement* or organic or inorganic))).ti,ab,kf. |
|  | 9 | lead/ or lead poisoning/ or exp organolead compound/ |
|  | 10 | lead.ti,kf,hw. or (pb or 208Pb or organolead* or plumbum or plumbic* or plumbate* or plumbous or tetraethyllead or (lead adj8 ("208" or metal* or organometal* or cation* or ion* or isotop* or radioisotop* or element* or microelement* or organic or inorganic or ore or blood* or chemical? or poison* or contam*))).ti,ab,kf. |
|  | 11 | arsenic/ or arsenic derivative/ or arsenic poisoning/ or exp arsine derivative/ or exp organoarsenic derivative/ |
|  | 12 | ((arsen$2 not arsenal) or arsen##$3 or arsine$1 or organoars*).ti,ab,kf. |
|  | 13 | cadmium/ or cadmium derivative/ or cadmium poisoning/ |
|  | 14 | (cadmi* or organocadmi* or (Cd and (metal* or organometal* or cation* or ion* or isotop* or radioisotop* or element* or microelement* or organic or inorganic))).ti,ab,kf. |
|  | 15 | exp perfluoroalkanoic acid/ or exp organofluorine derivative/ or polytetrafluoroethylene/ or freon/ |
|  | 16 | (perfluor* or polyfluor* or methylperfluor* or ethylperfluor* or fluorocarb* or fluorochemical* or fluorosurfactant* or fluorotelomer* or fluorinate* or PFAS$1 or PFBA$1 or PFBS$1 or PFC$1 or PFCA$1 or PFDA$1 or PFDoDA$1 or PFDS$1 or PFECHS$1 or PFESA$1 or PFEtCHxS$1 or PFHp$1 or PFHpA$1 or PFHpS$1 or PFHxA$1 or PFHxS$1 or PFNA$1 or PFNS$1 or PFOA$1 or PFOS$1 or PFOSA$1 or PFPA$1 or PFPeA$1 or PFPeS$1 or PFPiA$1 or PFSA$1 or PFTE$1 or PFTeDA$1 or PFTrDA$1 or PFUnDA$1 or FOSA$1 or EtFOSA$1 or MeFOSA$1 or DONA$1 or FTOH$1 or FTSA$1 or HFPO$1 or LCPFA$1 or SCPFA$1 or Teflon or freon or GenX).ti,ab,kf. |
|  | 17 | heavy metal/ or heavy metal blood level/ or heavy metal poisoning/ or (heavy adj metal*).ti,ab,kf. |
|  | 18 | or/7-17 |
| ***no animal studies*** | 19 | (exp animal/ not (exp animal/ and exp human/)) or nonhuman/ or exp animal experiment/ or exp experimental organism/ |
|  | 20 | ((animal? adj2 (study or model* or experiment*)) or mouse or mice or rat or rats or monkey or monkeys or "preclinical study").ti. |
|  | 21 | or/19-20 |
| **Protocol Question 2-6** | 22 | ((3 or 6) and 18) not 21 |
|  | 23 | ..l/ 22 en=y |

CAB Abstracts

Database: CAB Abstracts

Platform: Ovid

Date of search: April 2, 2025

Limits: English Language; Not animal studies

| Concept | Line | Search Strategy |
| --- | --- | --- |
| **human milk** | 1 | human milk/ |
|  | 2 | (((human? or breast* or maternal or mother*) adj milk*) or breastmilk? or colostrum).ti,ot,ab,id,hw. |
|  | 3 | or/1-2 |
| **infant formula** | 4 | infant formulae/ or bottle feeding/ or humanized milk/ |
|  | 5 | (((baby or babies or newborn* or "new born*" or infant* or neonat* or "neo nat*") adj formula*) or bottlefe* or (bottle adj (feed* or fed))).ti,ot,ab,id,hw. |
|  | 6 | or/4-5 |
| **lactation** | 7 | lactation/ or human lactation/ or exp lactating women/ or exp breast feeding/ |
|  | 8 | (lactat* or breastfe* or (breast adj (fed or feed*))).ti,ot,ab,id,hw. |
|  | 9 | or/7-8 |
| **contaminants** | 10 | mercury/ or exp mercury compounds/ or exp mercury fungicides/ |
|  | 11 | (mercur* or methylmercur* or organomercur* or MeHg or CH3Hg or (Hg and (metal* or organometal* or cation* or ion* or isotop* or radioisotop* or element* or microelement* or organic or inorganic))).ti,ot,ab,id,hw. |
|  | 12 | lead/ or lead poisoning/ |
|  | 13 | (lead or pb or 208Pb or organolead* or plumbum or plumbic* or plumbate* or plumbous or tetraethyllead or (lead adj8 ("208" or metal* or organometal* or cation* or ion* or isotop* or radioisotop* or element* or microelement* or organic or inorganic or ore or blood* or chemical? or poison* or contam*))).ti,ot,ab,id,hw. |
|  | 14 | arsenic/ or exp arsenicals/ |
|  | 15 | ((arsen$2 not arsenal) or arsen##$3 or arsine$1 or organoars*).ti,ot,ab,id,hw. |
|  | 16 | cadmium/ |
|  | 17 | (cadmi* or organocadmi* or (Cd and (metal* or organometal* or cation* or ion* or isotop* or radioisotop* or element* or microelement* or organic or inorganic))).ti,ot,ab,id,hw. |
|  | 18 | exp organofluorine compounds/ or exp organofluorine rodenticides/ or freons/ |
|  | 19 | (perfluor* or polyfluor* or methylperfluor* or ethylperfluor* or fluorocarb* or fluorochemical* or fluorosurfactant* or fluorotelomer* or fluorinate* or PFAS$1 or PFBA$1 or PFBS$1 or PFC$1 or PFCA$1 or PFDA$1 or PFDoDA$1 or PFDS$1 or PFECHS$1 or PFESA$1 or PFEtCHxS$1 or PFHp$1 or PFHpA$1 or PFHpS$1 or PFHxA$1 or PFHxS$1 or PFNA$1 or PFNS$1 or PFOA$1 or PFOS$1 or PFOSA$1 or PFPA$1 or PFPeA$1 or PFPeS$1 or PFPiA$1 or PFSA$1 or PFTE$1 or PFTeDA$1 or PFTrDA$1 or PFUnDA$1 or FOSA$1 or EtFOSA$1 or MeFOSA$1 or DONA$1 or FTOH$1 or FTSA$1 or HFPO$1 or LCPFA$1 or SCPFA$1 or Teflon or freon or GenX).ti,ot,ab,id,hw. |
|  | 20 | heavy metals/ or (heavy adj metal*).ti,ot,ab,id,hw. |
|  | 21 | or/10-20 |
| ***no animal studies*** | 22 | ((animals or plants or fungi or prokaryotes or viruses).sh,bt. not man/) or animal experiments/ or exp laboratory animals/ or animal models/ |
|  | 23 | ((animal? adj2 (study or model* or experiment*)) or mouse or mice or rat or rats or monkey or monkeys or "preclinical study").ti. |
|  | 24 | or/22-23 |
| **Protocol Question 2-6** | 25 | ((3 or 6 or 9) and 21) not 24 |
|  | 26 | limit 25 to English |

CINAHL

Database: CINAHL

Platform: EBSCO

Date of search: April 2, 2025

Limits: English Language; Not animal studies

Expanders: Apply equivalent subjects

| Concept | Line | Search Strategy |
| --- | --- | --- |
| **human milk** | S1 | (MH "Milk, Human+") or (MH "Milk Banks") |
|  | S2 | ((((TI human# OR AB human# OR SU human#) OR (TI breast* OR AB breast* OR SU breast*) OR (TI maternal OR AB maternal OR SU maternal) OR (TI mother* OR AB mother* OR SU mother*)) W1 (TI milk* OR AB milk* OR SU milk*)) OR (TI breastmilk# OR AB breastmilk# OR SU breastmilk#) OR (TI colostrum OR AB colostrum OR SU colostrum)) |
|  | S3 | S1 OR S2 |
| **infant formula** | S4 | (MH "Infant Formula+") |
|  | S5 | ((((TI baby OR AB baby OR SU baby) OR (TI babies OR AB babies OR SU babies) OR (TI newborn* OR AB newborn* OR SU newborn*) OR (TI "new born*" OR AB "new born*" OR SU "new born*") OR (TI infant* OR AB infant* OR SU infant*) OR (TI neonat* OR AB neonat* OR SU neonat*) OR (TI "neo nat*" OR AB "neo nat*" OR SU "neo nat*")) W1 (TI formula* OR AB formula* OR SU formula*)) OR (TI bottlefe* OR AB bottlefe* OR SU bottlefe*) OR ((TI bottle OR AB bottle OR SU bottle) W1 ((TI feed* OR AB feed* OR SU feed*) OR (TI fed OR AB fed OR SU fed)))) |
|  | S6 | S4 OR S5 |
| **lactation** | S7 | (MH "Lactation") or (MH "Infant Feeding+") or (MH "Infant Nutrition+") |
|  | S8 | ((TI lactat* OR AB lactat* OR SU lactat*) OR (TI breastfe* OR AB breastfe* OR SU breastfe*) OR ((TI breast OR AB breast OR SU breast) W1 ((TI fed OR AB fed OR SU fed) OR (TI feed* OR AB feed* OR SU feed*)))) |
|  | S9 | S7 OR S8 |
| **contaminants** | S10 | (MH "Mercury") or (MH "Mercury Poisoning") |
|  | S11 | ((TI mercur* OR AB mercur* OR SU mercur*) OR (TI methylmercur* OR AB methylmercur* OR SU methylmercur*) OR (TI organomercur* OR AB organomercur* OR SU organomercur*) OR (TI MeHg OR AB MeHg OR SU MeHg) OR (TI CH3Hg OR AB CH3Hg OR SU CH3Hg) OR ((TI Hg OR AB Hg OR SU Hg) AND ((TI metal* OR AB metal* OR SU metal*) OR (TI organometal* OR AB organometal* OR SU organometal*) OR (TI cation* OR AB cation* OR SU cation*) OR (TI ion* OR AB ion* OR SU ion*) OR (TI isotop* OR AB isotop* OR SU isotop*) OR (TI radioisotop* OR AB radioisotop* OR SU radioisotop*) OR (TI element* OR AB element* OR SU element*) OR (TI microelement* OR AB microelement* OR SU microelement*) OR (TI organic OR AB organic OR SU organic) OR (TI inorganic OR AB inorganic OR SU inorganic)))) |
|  | S12 | (MH "Lead") or (MH "Lead Exposure") or (MH "Lead Poisoning") |
|  | S13 | (TI lead,kf.) OR ((TI pb OR AB pb OR SU pb) OR (TI 208Pb OR AB 208Pb OR SU 208Pb) OR (TI organolead* OR AB organolead* OR SU organolead*) OR (TI plumbum OR AB plumbum OR SU plumbum) OR (TI plumbic* OR AB plumbic* OR SU plumbic*) OR (TI plumbate* OR AB plumbate* OR SU plumbate*) OR (TI plumbous OR AB plumbous OR SU plumbous) OR (TI tetraethyllead OR AB tetraethyllead OR SU tetraethyllead) OR ((TI lead OR AB lead OR SU lead) N8 ((TI 208 OR AB 208 OR SU 208) OR (TI metal* OR AB metal* OR SU metal*) OR (TI organometal* OR AB organometal* OR SU organometal*) OR (TI cation* OR AB cation* OR SU cation*) OR (TI ion* OR AB ion* OR SU ion*) OR (TI isotop* OR AB isotop* OR SU isotop*) OR (TI radioisotop* OR AB radioisotop* OR SU radioisotop*) OR (TI element* OR AB element* OR SU element*) OR (TI microelement* OR AB microelement* OR SU microelement*) OR (TI organic OR AB organic OR SU organic) OR (TI inorganic OR AB inorganic OR SU inorganic) OR (TI ore OR AB ore OR SU ore) OR (TI blood* OR AB blood* OR SU blood*) OR (TI chemical# OR AB chemical# OR SU chemical#) OR (TI poison* OR AB poison* OR SU poison*) OR (TI contam* OR AB contam* OR SU contam*)))) |
|  | S14 | (MH "Arsenic") or (MH "Arsenicals+") or (MH "Arsenic Poisoning") |
|  | S15 | (((TI arsen* OR AB arsen* OR SU arsen*) NOT (TI arsenal OR AB arsenal OR SU arsenal)) OR (TI arsine# OR AB arsine# OR SU arsine#) OR (TI organoars* OR AB organoars* OR SU organoars*)) |
|  | S16 | (MH "Cadmium") or (MH "Cadmium Compounds+") |
|  | S17 | ((TI cadmi* OR AB cadmi* OR SU cadmi*) OR (TI organocadmi* OR AB organocadmi* OR SU organocadmi*) OR ((TI Cd OR AB Cd OR SU Cd) AND ((TI metal* OR AB metal* OR SU metal*) OR (TI organometal* OR AB organometal* OR SU organometal*) OR (TI cation* OR AB cation* OR SU cation*) OR (TI ion* OR AB ion* OR SU ion*) OR (TI isotop* OR AB isotop* OR SU isotop*) OR (TI radioisotop* OR AB radioisotop* OR SU radioisotop*) OR (TI element* OR AB element* OR SU element*) OR (TI microelement* OR AB microelement* OR SU microelement*) OR (TI organic OR AB organic OR SU organic) OR (TI inorganic OR AB inorganic OR SU inorganic)))) |
|  | S18 | (MH “Hydrocarbons, Fluorinated+”) |
|  | S19 | ((TI perfluor* OR AB perfluor* OR SU perfluor*) OR (TI polyfluor* OR AB polyfluor* OR SU polyfluor*) OR (TI methylperfluor* OR AB methylperfluor* OR SU methylperfluor*) OR (TI ethylperfluor* OR AB ethylperfluor* OR SU ethylperfluor*) OR (TI fluorocarb* OR AB fluorocarb* OR SU fluorocarb*) OR (TI fluorochemical* OR AB fluorochemical* OR SU fluorochemical*) OR (TI fluorosurfactant* OR AB fluorosurfactant* OR SU fluorosurfactant*) OR (TI fluorotelomer* OR AB fluorotelomer* OR SU fluorotelomer*) OR (TI fluorinate* OR AB fluorinate* OR SU fluorinate*) OR (TI PFAS# OR AB PFAS# OR SU PFAS#) OR (TI PFBA# OR AB PFBA# OR SU PFBA#) OR (TI PFBS# OR AB PFBS# OR SU PFBS#) OR (TI PFC# OR AB PFC# OR SU PFC#) OR (TI PFCA# OR AB PFCA# OR SU PFCA#) OR (TI PFDA# OR AB PFDA# OR SU PFDA#) OR (TI PFDoDA# OR AB PFDoDA# OR SU PFDoDA#) OR (TI PFDS# OR AB PFDS# OR SU PFDS#) OR (TI PFECHS# OR AB PFECHS# OR SU PFECHS#) OR (TI PFESA# OR AB PFESA# OR SU PFESA#) OR (TI PFEtCHxS# OR AB PFEtCHxS# OR SU PFEtCHxS#) OR (TI PFHp# OR AB PFHp# OR SU PFHp#) OR (TI PFHpA# OR AB PFHpA# OR SU PFHpA#) OR (TI PFHpS# OR AB PFHpS# OR SU PFHpS#) OR (TI PFHxA# OR AB PFHxA# OR SU PFHxA#) OR (TI PFHxS# OR AB PFHxS# OR SU PFHxS#) OR (TI PFNA# OR AB PFNA# OR SU PFNA#) OR (TI PFNS# OR AB PFNS# OR SU PFNS#) OR (TI PFOA# OR AB PFOA# OR SU PFOA#) OR (TI PFOS# OR AB PFOS# OR SU PFOS#) OR (TI PFOSA# OR AB PFOSA# OR SU PFOSA#) OR (TI PFPA# OR AB PFPA# OR SU PFPA#) OR (TI PFPeA# OR AB PFPeA# OR SU PFPeA#) OR (TI PFPeS# OR AB PFPeS# OR SU PFPeS#) OR (TI PFPiA# OR AB PFPiA# OR SU PFPiA#) OR (TI PFSA# OR AB PFSA# OR SU PFSA#) OR (TI PFTE# OR AB PFTE# OR SU PFTE#) OR (TI PFTeDA# OR AB PFTeDA# OR SU PFTeDA#) OR (TI PFTrDA# OR AB PFTrDA# OR SU PFTrDA#) OR (TI PFUnDA# OR AB PFUnDA# OR SU PFUnDA#) OR (TI FOSA# OR AB FOSA# OR SU FOSA#) OR (TI EtFOSA# OR AB EtFOSA# OR SU EtFOSA#) OR (TI MeFOSA# OR AB MeFOSA# OR SU MeFOSA#) OR (TI DONA# OR AB DONA# OR SU DONA#) OR (TI FTOH# OR AB FTOH# OR SU FTOH#) OR (TI FTSA# OR AB FTSA# OR SU FTSA#) OR (TI HFPO# OR AB HFPO# OR SU HFPO#) OR (TI LCPFA# OR AB LCPFA# OR SU LCPFA#) OR (TI SCPFA# OR AB SCPFA# OR SU SCPFA#) OR (TI Teflon OR AB Teflon OR SU Teflon) OR (TI freon OR AB freon OR SU freon) OR (TI GenX OR AB GenX OR SU GenX)) |
|  | S20 | (MH "Metals, Heavy") OR (MH "Heavy Metal Poisoning") OR ((TI heavy OR AB heavy OR SU heavy) W1 (TI metal* OR AB metal* OR SU metal*)) |
|  | S21 | S10 OR S11 OR S12 OR S13 OR S14 OR S15 OR S16 OR S17 OR S18 OR S19 OR S20 |
| ***no animal studies*** | S22 | ((MH Animals+) NOT ((MH Animals+) AND (MH Humans))) OR (MH "Animal Experimentation+") OR (MH "Models, Animal+") |
|  | S23 | (((TI animal#) N2 ((TI study) OR (TI model*) OR (TI experiment*))) OR (TI mouse) OR (TI mice) OR (TI rat) OR (TI rats) OR (TI monkey) OR (TI monkeys) OR (TI "preclinical study")) |
|  | S24 | S22 OR S24 |
| **Protocol Question 2-6** | S25 | ((S3 OR S6 OR S9) AND S21) NOT S24 |
|  | S26 | S25 AND (LA english) |

CENTRAL

Database: CENTRAL

Platform: Cochrane Library

Date of search: April 2, 2025

Limits: English Language; Not animal studies

| Concept | Line | | Search Strategy |
| --- | --- | --- | --- |
| **human milk** | | #1 | [mh "Milk, Human"] |
|  |  | #2 | (((human?:ti,ab,kw OR breast*:ti,ab,kw OR maternal:ti,ab,kw OR mother*:ti,ab,kw) NEXT milk*:ti,ab,kw) OR breastmilk?:ti,ab,kw OR colostrum:ti,ab,kw) |
|  |  | #3 | #1 OR #2 |
| **infant formula** | | #4 | [mh "Infant Formula"] OR [mh "Bottle Feeding"] |
|  |  | #5 | (((baby:ti,ab,kw OR babies:ti,ab,kw OR newborn*:ti,ab,kw OR ("new" NEXT born*):ti,ab,kw OR infant*:ti,ab,kw OR neonat*:ti,ab,kw OR ("neo" NEXT nat*):ti,ab,kw) NEXT formula*:ti,ab,kw) OR bottlefe*:ti,ab,kw OR (bottle:ti,ab,kw NEXT (feed*:ti,ab,kw OR fed:ti,ab,kw))) |
|  |  | #6 | #4 OR #5 |
| **lactation** | | #7 | [mh Lactation] OR [mh "Breast Feeding"] |
|  | | #8 | (lactat*:ti,ab,kw OR breastfe*:ti,ab,kw OR (breast:ti,ab,kw NEXT (fed:ti,ab,kw OR feed*:ti,ab,kw))) |
|  |  | #9 | #7 OR #8 |
| **contaminants** | | #10 | [mh Mercury] OR [mh "Mercury Compounds"] OR [mh "Organomercury Compounds"] OR [mh "Mercury Poisoning"] |
|  |  | #11 | (mercur*:ti,ab,kw OR methylmercur*:ti,ab,kw OR organomercur*:ti,ab,kw OR MeHg:ti,ab,kw OR CH3Hg:ti,ab,kw OR (Hg:ti,ab,kw AND (metal*:ti,ab,kw OR organometal*:ti,ab,kw OR cation*:ti,ab,kw OR ion*:ti,ab,kw OR isotop*:ti,ab,kw OR radioisotop*:ti,ab,kw OR element*:ti,ab,kw OR microelement*:ti,ab,kw OR organic:ti,ab,kw OR inorganic:ti,ab,kw))) |
|  |  | #12 | [mh "Lead Poisoning"] OR [mh Lead] OR [mh "Lead Radioisotopes"] OR [mh "Tetraethyl Lead"] |
|  |  | #13 | lead:ti,kw OR (pb:ti,ab,kw OR 208Pb:ti,ab,kw OR organolead*:ti,ab,kw OR plumbum:ti,ab,kw OR plumbic*:ti,ab,kw OR plumbate*:ti,ab,kw OR plumbous:ti,ab,kw OR tetraethyllead:ti,ab,kw OR (lead:ti,ab,kw NEAR/8 (208:ti,ab,kw OR metal*:ti,ab,kw OR organometal*:ti,ab,kw OR cation*:ti,ab,kw OR ion*:ti,ab,kw OR isotop*:ti,ab,kw OR radioisotop*:ti,ab,kw OR element*:ti,ab,kw OR microelement*:ti,ab,kw OR organic:ti,ab,kw OR inorganic:ti,ab,kw OR ore:ti,ab,kw OR blood*:ti,ab,kw OR chemical?:ti,ab,kw OR poison*:ti,ab,kw OR contam*:ti,ab,kw))) |
|  |  | #14 | [mh "Arsenic Poisoning"] OR [mh Arsenic] OR [mh Arsenicals] |
|  |  | #15 | ((arsen*:ti,ab,kw NOT arsenal:ti,ab,kw) OR arsine?:ti,ab,kw OR organoars*:ti,ab,kw) |
|  |  | #16 | [mh Cadmium] OR [mh "Cadmium Poisoning"] OR [mh "Cadmium Radioisotopes"] OR [mh "Cadmium Compounds"] |
|  |  | #17 | (cadmi*:ti,ab,kw OR organocadmi*:ti,ab,kw OR (Cd:ti,ab,kw AND (metal*:ti,ab,kw OR organometal*:ti,ab,kw OR cation*:ti,ab,kw OR ion*:ti,ab,kw OR isotop*:ti,ab,kw OR radioisotop*:ti,ab,kw OR element*:ti,ab,kw OR microelement*:ti,ab,kw OR organic:ti,ab,kw OR inorganic:ti,ab,kw))) |
|  |  | #18 | [mh Fluorocarbons] |
|  |  | #19 | (perfluor*:ti,ab,kw OR polyfluor*:ti,ab,kw OR methylperfluor*:ti,ab,kw OR ethylperfluor*:ti,ab,kw OR fluorocarb*:ti,ab,kw OR fluorochemical*:ti,ab,kw OR fluorosurfactant*:ti,ab,kw OR fluorotelomer*:ti,ab,kw OR fluorinate*:ti,ab,kw OR PFAS?:ti,ab,kw OR PFBA?:ti,ab,kw OR PFBS?:ti,ab,kw OR PFC?:ti,ab,kw OR PFCA?:ti,ab,kw OR PFDA?:ti,ab,kw OR PFDoDA?:ti,ab,kw OR PFDS?:ti,ab,kw OR PFECHS?:ti,ab,kw OR PFESA?:ti,ab,kw OR PFEtCHxS?:ti,ab,kw OR PFHp?:ti,ab,kw OR PFHpA?:ti,ab,kw OR PFHpS?:ti,ab,kw OR PFHxA?:ti,ab,kw OR PFHxS?:ti,ab,kw OR PFNA?:ti,ab,kw OR PFNS?:ti,ab,kw OR PFOA?:ti,ab,kw OR PFOS?:ti,ab,kw OR PFOSA?:ti,ab,kw OR PFPA?:ti,ab,kw OR PFPeA?:ti,ab,kw OR PFPeS?:ti,ab,kw OR PFPiA?:ti,ab,kw OR PFSA?:ti,ab,kw OR PFTE?:ti,ab,kw OR PFTeDA?:ti,ab,kw OR PFTrDA?:ti,ab,kw OR PFUnDA?:ti,ab,kw OR FOSA?:ti,ab,kw OR EtFOSA?:ti,ab,kw OR MeFOSA?:ti,ab,kw OR DONA?:ti,ab,kw OR FTOH?:ti,ab,kw OR FTSA?:ti,ab,kw OR HFPO?:ti,ab,kw OR LCPFA?:ti,ab,kw OR SCPFA?:ti,ab,kw OR Teflon:ti,ab,kw OR freon:ti,ab,kw OR GenX:ti,ab,kw) |
|  |  | #20 | [mh ^"Metals, Heavy"] OR [mh ^"Heavy Metal Poisoning"] OR (heavy:ti,ab,kw NEXT metal*:ti,ab,kw) |
|  | | #21 | or/10-20 |
| ***no animal studies*** | | #22 | ([mh Animals] NOT ([mh Animals] AND [mh ^Humans])) OR [mh "Animal Experimentation"] OR [mh "Models, Animal"] |
|  |  | #23 | ((animal?:ti NEAR/2 (study:ti OR model*:ti OR experiment*:ti)) OR mouse:ti OR mice:ti OR rat:ti OR rats:ti OR monkey:ti OR monkeys:ti OR "preclinical study":ti) |
|  |  | #24 | #22 OR #23 |
| **Protocol Question 2-6** | | #25 | ((#3 OR #6 OR #9) AND #21) not #24 |
|  |  | #26 | #25 AND english:la |

*Journal of Environmental Exposure Assessment*

Platform: https://www.oaepublish.com/jeea

Date of search: April 21, 2025

Limits: None

| **Key terms searched in the journal’s search engine one at a time** | **Results** |
| --- | --- |
| human milk | 4 |
| infant formula | 3 |
| infant | 5 (all duplicates) |
| arsenic | 2 |
| cadmium | 1 |
| lead | 0 |
| mercury | 1 |
| PFAS | 2 (1 duplicate) |
| per- and polyfluoroalkyl substances | 1 (duplicate) |
| metal | 1 |
| heavy metals | 0 |
| breastmilk | 2 (all duplicates) |
| breast milk | 2 (all duplicates) |
| baby | 2 (all duplicates) |

## **Supplemental Table 3.** Inclusion and exclusion criteria for ‘What are biospecimen concentrations of arsenic, cadmium, lead, mercury, or PFAS of infants living in the United States that are consuming human milk and/or infant formula?’ (PROSPERO: CRD42024530344).

| Criterion | Include | Exclude |
| --- | --- | --- |
| Population | General infants aged ≤12 months  Studies that enroll some infants who are born preterm (<37 weeks and 0/7 days gestational age) | Studies that ***exclusively*** enroll infants diagnosed with a disease or developmental disorder or hospitalized with an illness or injury.  Studies that ***exclusively*** enroll infants who are born preterm (<37 weeks and 0/7 days gestational age)  Non-human studies including animal studies, cell models, simulation or modeling studies |
| Exposure | N/A | N/A |
| Comparator | N/A | N/A |
| Outcome | Measured concentrations of contaminants in infant biospecimens in relation to the consumption of human milk and/or infant formula  Contaminants of interest: As (total and inorganic), Pb, Cd, Hg (total and methylmercury), PFAS | Data or analyses not reported based on infant feeding (human milk only, infant formula only, or human milk and infant formula)  Measures from cord blood, meconium, or hair collected at birth as this would have been taken prior to infant consumption of human milk or infant formula |
| Setting | United States | Outside of the US |
| Study design | Randomized controlled trials  Non-randomized controlled trials and quasi-experimental studies  Prospective or retrospective cohort studies  Cross-sectional studies  Descriptive studies and other designs without a comparison group | Modeling/simulation studies  Case-control studies  Literature reviews, including systematic reviews and meta-analyses |
| Publication status | Peer-reviewed studies published in scholarly journals | Other content published in scholarly journals (e.g., conference abstracts, letters to the editor, commentaries)  Dissertations/theses  Grey literature |
| Date range | N/A | N/A |
| Language | English | Other languages |

N/A, not applicable.

## **Supplemental Table 4.** Inclusion and exclusion criteria for ‘What is the bioavailability of arsenic, cadmium, lead, mercury, or PFAS from human milk and infant formula when consumed by the infant?’ (PROSPERO: CRD42024530332).

| Criterion | Include | Exclude |
| --- | --- | --- |
| Population | General infants aged ≤12 months  Studies that enroll some infants who are born preterm (<37 weeks and 0/7 days gestational age) | Studies that ***exclusively*** enroll infants diagnosed with a disease or developmental disorder or hospitalized with an illness or injury.  Studies that ***exclusively*** enroll infants who are born preterm (<37 weeks and 0/7 days gestational age)  Non-human studies including animal studies, cell models, simulation or modeling studies  Studies that include infants who have been fed complementary foods and beverages |
| Exposure | Measured concentrations of contaminants in human milk and/or infant formula being consumed by an infant  Contaminants of interest: As (total and inorganic), Pb, Cd, Hg (total and methylmercury), PFAS | Does not measure concentration of contaminant in human milk and/or infant formula being consumed by an infant  General/population-level measurement or estimate |
| Comparator | N/A | N/A |
| Outcome | Bioavailability (ratio of contaminant level in infant to contaminant levels in human milk and/or infant formula consumed by the infant) | No measure of bioavailability |
| Setting | Countries rated high or very high on the Human Development Index^a^ | Countries rated low or medium on the Human Development Index^a^ |
| Study design | Randomized controlled trials  Non-randomized controlled trials and quasi-experimental studies  Prospective or retrospective cohort studies  Cross-sectional studies  Descriptive studies and other designs without a comparison group | Modeling/simulation studies  Case-control studies  Literature reviews, including systematic reviews and meta-analyses |
| Publication status | Peer-reviewed studies published in scholarly journals | Other content published in scholarly journals (e.g., conference abstracts, letters to the editor, commentaries)  Dissertations/theses  Grey literature |
| Date range | N/A | N/A |
| Language | English | Other languages |

aHuman Development Index (HDI) is a “summary measure of average achievement in key dimensions of human development: a long and healthy life, being knowledgeable and having a decent standard of living.” <https://hdr.undp.org/data-center/human-development-index>

## **Supplemental Table 5.** Inclusion and exclusion criteria for ‘What factors impact the bioavailability of arsenic, cadmium, lead, mercury, or PFAS from human milk and infant formula when consumed by the infant?’ (PROSPERO: CRD42024530336).

| Criterion | Include | Exclude |
| --- | --- | --- |
| Population | General infants aged ≤12 months  Studies that enroll some infants who are born preterm (<37 weeks and 0/7 days gestational age) | Studies that ***exclusively*** enroll infants diagnosed with a disease or developmental disorder or hospitalized with an illness or injury.  Studies that ***exclusively*** enroll infants who are born preterm (<37 weeks and 0/7 days gestational age)  Non-human studies including animal studies, cell models, simulation or modeling studies |
| Exposure | Measured concentrations of contaminants in human milk and/or infant formula being consumed by an infant; Contaminants of interest: As (total and inorganic), Pb, Cd, Hg (total and methylmercury), PFAS  Measured level of “other factor” (e.g., vitamin, mineral, nutrient, other contaminants) | Does not measure concentrations of contaminant in human milk and/or infant formula being consumed by an infant  No measurement of “other factor”  General/Population-level measurement or estimate |
| Comparator | Different amounts of the exposure to the “other” factor | No measure of contaminant or “other” exposure |
| Outcome | Bioavailability (ratio of contaminant level in infant to contaminant levels in human milk or infant formula consumed by the infant) | No measure of bioavailability |
| Setting | Countries rated high or very high on the Human Development Index^a^ | Countries rated low or medium on the Human Development Index^a^ |
| Study design | Randomized controlled trials  Non-randomized controlled trials and quasi-experimental studies  Prospective or retrospective cohort studies  Cross-sectional studies | Descriptive studies and other designs without a comparison group  Modeling/simulation studies  Case-control studies  Literature reviews, including systematic reviews and meta-analyses |
| Publication status | Peer-reviewed studies published in scholarly journals | Other content published in scholarly journals (e.g., conference abstracts, letters to the editor, commentaries)  Dissertations/theses  Grey literature |
| Date range | N/A | N/A |
| Language | English | Other languages |

aHuman Development Index (HDI) is a “summary measure of average achievement in key dimensions of human development: a long and healthy life, being knowledgeable and having a decent standard of living.”

<https://hdr.undp.org/data-center/human-development-index>

## **Supplemental Table 6.** Inclusion and exclusion criteria for ‘What is the relationship between arsenic, cadmium, lead, mercury, or PFAS and the bioavailability of other components in human milk and infant formula?’ (PROSPERO: CRD42024530339).

| Criterion | Include | Exclude |
| --- | --- | --- |
| Population | General infants aged ≤12 months  Studies that enroll some infants who are born preterm (<37 weeks and 0/7 days gestational age) | Studies that ***exclusively*** enroll infants diagnosed with a disease or developmental disorder or hospitalized with an illness or injury.  Studies that ***exclusively*** enroll infants who are born preterm (<37 weeks and 0/7 days gestational age)  Non-human studies including animal studies, cell models, simulation or modeling studies |
| Exposure | Measured concentrations of contaminants in human milk and infant formula being consumed by an infant;  Contaminants of interest: As (total and inorganic), Pb, Cd, Hg (total and methylmercury), PFAS | Does not measure concentrations of contaminant in human milk and infant formula being consumed by an infant  General/population-level measurement or estimate |
| Comparator | Different amounts of the exposure | No measure of exposure |
| Outcome | Bioavailability of “other component” of human milk or infant formula (ratio of “other component” levels in infants to “other component” levels in human milk or infant formula being consumed by infant)  AND  Analysis of modification effect of contaminant on the bioavailability of the “other component” | No measure of bioavailability  No analysis of effect modification by the contaminant |
| Setting | Countries rated high or very high on the Human Development Index^a^ | Countries rated low or medium on the Human Development Index^a^ |
| Study design | Randomized controlled trials  Non-randomized controlled trials and quasi-experimental studies  Prospective or retrospective cohort studies  Cross-sectional studies | Descriptive studies and other designs without a comparison group  Modeling/simulation studies  Case-control studies  Literature reviews, including systematic reviews and meta-analyses |
| Publication status | Peer-reviewed studies published in scholarly journals | Other content published in scholarly journals (e.g., conference abstracts, letters to the editor, commentaries)  Dissertations/theses  Grey literature |
| Date range | N/A | N/A |
| Language | English | Other languages |

aHuman Development Index (HDI) is a “summary measure of average achievement in key dimensions of human development: a long and healthy life, being knowledgeable and having a decent standard of living.”

<https://hdr.undp.org/data-center/human-development-index>

**Supplemental Table 7.** Operationalization of ROBINS-E^a^ for ‘What are biospecimen concentrations of arsenic, cadmium, lead, mercury, or PFAS of infants living in the United States that are consuming human milk and/or infant formula?’ (PROSPERO: CRD42024530344).

| **Domains** | **ROBINS-E Signaling Questions** | **Operationalization** |
| --- | --- | --- |
| **Domain1. Risk of bias due to confounding** | No change; studies were considered at low risk because there were no confounders in the analytical framework (Supplemental Figure 1) identified by the Technical Expert Panel. | |
| **Domain 2: Risk of bias arising from measurement of the exposure** | No change; studies were considered at low risk because the exposure was not considered to contribute to bias based on the analytical framework (Supplemental Figure 1) informed by the Technical Expert Panel. | |
| **Domain 3: Risk of bias in selection of participants into the study (or into the analysis)** | 3.1 Did follow-up begin at (or close to) the start of the exposure window for most participants?”  Guidance: For exposures known to have a long latency period (i.e. outcomes that are impacted by the exposure are not expected to occur until after this period), bias is unlikely to be introduced if follow-up starts during the latency period for most participants. In this situation, it is reasonable to answer ‘Yes’ or ‘Probably Yes’ to this question. | Operationalized: ‘Yes’ or “Probably Yes’ for cross-sectional studies; contaminant exposure is likely to have a long latency period. |
|  | 3.3 Was selection of participants into the study (or into the analysis) based on participant characteristics observed after the start of the exposure window being studied? | Operationalized for cross-sectional studies: Was selection of participants into the study (or into the analysis) based on participant characteristics? |
| **Domain 4: Risk of bias due to post-exposure interventions** | No change; studies were considered at low risk if they were assessing lifetime contaminant exposure in a general population, in which a reason for intervention would be unlikely. Studies that measured contaminant exposure related to an environmental or poisoning event in which an intervention may be expected before measurement of the outcome were handled on a case-by-case basis. | |
| **Domain 5: Risk of bias due to missing data** | 5.1 Were complete data on exposure status available for all, or nearly all, participants?  5.3 Were complete data on confounding variables available for all, or nearly all, participants? | Operationalized: ‘Yes’ or ‘Probably Yes’ for all studies because confounding and exposure were not considered as influential sources of bias based on the analytical framework developed by the Technical Expert Panel. |
| **Domain 6: Risk of bias arising from measurement of outcomes** | No changes. | |
| **Domain 7: Risk of bias in selection of the reported result** | No changes. | |

^a^Table only includes items that were operationalized in a specific way based on the analytical framework for the research question, informed by the Technical Expert Panel. For full list of domains and signaling questions, please visit: <https://www.riskofbias.info/welcome/robins-e-tool>. No articles were identified for the questions related to bioavailability so risk of bias was not assessed for those questions.

## **Supplemental Table 8.** Excluded full text articles identified from the database search, backwards citation search, or manual search of the *Jornal of Environmental Exposure Assessment* with reasons for exclusion.

| **Question Topic** | **Article** | **Reason for Exclusion** |
| --- | --- | --- |
| Infant exposure to contaminants from HM and/or IF | Galster, W A. Mercury in Alaskan Eskimo mothers and infants. Environmental health perspectives 15 (1976): 135-40 | Outcome  Cord blood (ineligible as an infant biomarker because exposure to human milk or infant formula did not precede biospecimen collection). |
| Infant exposure to contaminants from HM and/or IF | Bauer, Julia A; Punshon, Tracy; Barr, Matthew N; Jackson, Brian P; Weisskopf, Marc G; Bidlack, Felicitas B; Coker, Modupe O; Peacock, Janet L; Karagas, Margaret R. Deciduous teeth from the New Hampshire birth cohort study: Early life environmental and dietary predictors of dentin elements. Environmental research 256 (2024): 119170 | Population  Teeth were “naturally exfoliated” which occurs in childhood. Therefore, the measurement occurred after the exposure window of interest (≤ 12 months). |
| Infant exposure to contaminants from HM and/or IF | Ziegler, E.; Edwards, B.; Jensen, R.; Mahaffey, K.; Fomon, S. Absorption and Retention of Lead by Infants. Pediatric Research 12 (1978): 29–34 | Population^a^  Infant age ranged from 14 to 746 days and could not be stratified by those ≤ and > 12 months. Therefore, the measurement occurred after the exposure window of interest  (≤ 12 months). |
| Infant exposure to contaminants from HM and/or IF | Signes-Pastor, Antonio J; Cottingham, Kathryn L; Carey, Manus; Sayarath, Vicki; Palys, Thomas; Meharg, Andrew A; Folt, Carol L; Karagas, Margaret R. Infants’ dietary arsenic exposure during transition to solid food. Scientific reports 8 (2018): 7114 | Duplicate record with database search^a^ |
| Infant exposure to contaminants from HM and/or IF | Andjelkovic, M.; Van Overmeire, I.; Joly, L.; Poma, G.; Malarvannan, G.; Vleminckx, C.; Malysheva, S. V.; Vanhouche, M.; Van Loco, J.; Van Nieuwenhuyse, A.; Covaci, A. Persistent organic pollutants in human milk of Belgian mothers: levels, time trend and exposure assessment for nursing infants. Journal of Environmental Exposure Assessment 3 (2024): 23 | Outcome^b^  Persistent organic pollutants |
| Bioavailability | Linares, Ana Maria; Thaxton-Wiggins, Amanda; Unrine, Jason M. Concentrations of Lead and Arsenic in Mother’s Milk and Children’s Blood in Peruvian Breastfeeding Dyads. Journal of human lactation: official journal of International Lactation Consultant Association 40 (2024): 69-79 | Bioavailability not assessed |
| Bioavailability | Yao, Jingzhi; Dong, Zhaomin; Jiang, Lulin; Pan, Yitao; Zhao, Meirong; Bai, Xiaoxia; Dai, Jiayin. Emerging and Legacy Perfluoroalkyl Substances in Breastfed Chinese Infants: Renal Clearance, Body Burden, and Implications. Environmental health perspectives 131 (2023): 37003 | Bioavailability not assessed |
| Bioavailability | Garcia Salcedo, Jose Javier; Roh, Taehyun; Nava Rivera, Lydia Enith; Betancourt Martinez, Nadia Denys; Carranza Rosales, Pilar; San Miguel Salazar, Maria Francisco; Rivera Guillen, Mario Alberto; Serrano Gallardo, Luis Benjamin; Nino Castaneda, Maria Sonadora; Guzman Delgado, Nacny Elena; Millan Orozco, Jair; Ortega Morales, Natalia; Moran Martinez, Javier. Comparative Biomonitoring of Arsenic Exposure in Mothers and Their Neonates in Comarca Lagunera, Mexico. International journal of environmental research and public health 19 (2022): 16232 | Study design |
| Bioavailability | van Beijsterveldt, Inge A L P; van Zelst, Bertrand D; de Fluiter, Kirsten S; van den Berg, Sjoerd A A; van der Steen, Manouk; Hokken-Koelega, Anita C S. Poly- and perfluoroalkyl substances (PFAS) exposure through infant feeding in early life. Environment international 164 (2022): 107274 | Bioavailability not assessed |
| Bioavailability | Orun, Emel; Yalcin, S Songul; Aykut, Osman. Lead, mercury, and cadmium levels in breast milk and infant hair in the late period of lactation in Ankara, Turkey. International journal of environmental health research 32 (2022): 1950-1961 | Study design |
| Bioavailability | Al-Saleh, Iman; Moncari, Lina; Jomaa, Ahmed; Elkhatib, Rola; Al-Rouqi, Reem; Eltabache, Chafica; Al-Rajudi, Tahreer; Alnuwaysir, Hissah; Nester, Michael; Aldhalaan, Hesham. Effects of early and recent mercury and lead exposure on the neurodevelopment of children with elevated mercury and/or developmental delays during lactation: A follow-up study. International journal of hygiene and environmental health 230 (2020): 113629 | Population  Study *exclusively* recruits infants with developmental disorders |
| Bioavailability | Al-Saleh, Iman; Elkhatib, Rola; Al-Rouqi, Reem; Abduljabbar, Mai; Eltabache, Chafica; Al-Rajudi, Tahreer; Nester, Michael. Alterations in biochemical markers due to mercury (Hg) exposure and its influence on infant's neurodevelopment. International journal of hygiene and environmental health 219 (2016): 898-914 | Bioavailability not assessed |
| Bioavailability | Al-Saleh, Iman; Nester, Michael; Abduljabbar, Mai; Al-Rouqi, Reem; Eltabache, Chafica; Al-Rajudi, Tahreer; Elkhatib, Rola. Mercury (Hg) exposure and its effects on Saudi breastfed infant's neurodevelopment. International journal of hygiene and environmental health 219 (2016): 129-41 | Duplicate data  Duplicate data with Al-Saleh, et al. Int J Hyg Environ Health (2016) |
| Bioavailability | Al-Saleh, Iman; Abduljabbar, Mai; Al-Rouqi, Reem; Eltabache, Chafica; Al-Rajudi, Tahreer; Elkhatib, Rola; Nester, Michael. The extent of mercury (Hg) exposure among Saudi mothers and their respective infants. Environmental monitoring and assessment 187 (2015): 678 | Duplicate data  Duplicate data with Al-Saleh, et al. Int J Hyg Environ Health (2016) |
| Bioavailability | Al-Saleh, Iman; Abduljabbar, Mai; Al-Rouqi, Reem; Elkhatib, Rola; Alshabbaheen, Ammar; Shinwari, Neptune. Mercury (Hg) exposure in breast-fed infants and their mothers and the evidence of oxidative stress. Biological trace element research 153 (2013): 145-54 | Duplicate data  Duplicate data with Al-Saleh, et al. Int J Hyg Environ Health (2016) |
| Bioavailability | Astolfi, Maria Luisa; Protano, Carmela; Schiavi, Elisa; Marconi, Elisabetta; Capobianco, Daniela; Massimi, Lorenzo; Ristorini, Martina; Baldassarre, Maria Elisabetta; Laforgia, Nicola; Vitali, Matteo; Canepari, Silvia; Mastromarino, Paola. A prophylactic multi-strain probiotic treatment to reduce the absorption of toxic elements: In-vitro study and biomonitoring of breast milk and infant stools. Environment international 130 (2019): 104818 | Bioavailability not assessed |
| Bioavailability | Olszowski, Tomasz; Baranowska-Bosiacka, Irena; Rebacz-Maron, Ewa; Gutowska, Izabela; Jamiol, Dominika; Prokopowicz, Adam; Goschorska, Marta; Chlubek, Dariusz. Cadmium Concentration in Mother's Blood, Milk, and Newborn's Blood and Its Correlation with Fatty Acids, Anthropometric Characteristics, and Mother's Smoking Status. Biological trace element research 174 (2016): 8-20 | Bioavailability not assessed |
| Bioavailability | Dursun, Arzu; Yurdakok, Kadriye; Yalcin, Songul S; Tekinalp, Gulsevin; Aykut, Osman; Orhan, Gunnur; Morgil, Goksel Koc. Maternal risk factors associated with lead, mercury and cadmium levels in umbilical cord blood, breast milk and newborn hair. The journal of maternal-fetal & neonatal medicine: the official journal of the European Association of Perinatal Medicine, the Federation of Asia and Oceania Perinatal Societies, the International Society of Perinatal Obstetricians 29 (2016): 954-61 | Bioavailability not assessed |
| Bioavailability | Carignan, Courtney C; Cottingham, Kathryn L; Jackson, Brian P; Farzan, Shohreh F; Gandolfi, A Jay; Punshon, Tracy; Folt, Carol L; Karagas, Margaret R. Estimated exposure to arsenic in breastfed and formula-fed infants in a United States cohort. Environmental health perspectives 123 (2015): 500-6 | Study design |
| Bioavailability | Castro, Francisca; Harari, Florencia; Llanos, Miguel; Vahter, Marie; Ronco, Ana Maria. Maternal-child transfer of essential and toxic elements through breast milk in a mine-waste polluted area. American journal of perinatology 31 (2014): 993-1002 | Study design |
| Bioavailability | Marques, Rejane C; Bernardi, Jose V E; Dorea, Jose G; de Fatima R Moreira, Maria; Malm, Olaf. Perinatal multiple exposure to neurotoxic (lead, methylmercury, ethylmercury, and aluminum) substances and neurodevelopment at six and 24 months of age. Environmental pollution (Barking, Essex: 1987) 187 (2014): 130-5 | Bioavailability not assessed |
| Bioavailability | Deroma, L; Parpinel, M; Tognin, V; Channoufi, L; Tratnik, J; Horvat, M; Valent, F; Barbone, F. Neuropsychological assessment at school-age and prenatal low-level exposure to mercury through fish consumption in an Italian birth cohort living near a contaminated site. International journal of hygiene and environmental health 216 (2013): 486-93 | Bioavailability not assessed |
| Bioavailability | Sakamoto, Mineshi; Chan, Hing Man; Domingo, Jose L; Kubota, Machi; Murata, Katsuyuki. Changes in body burden of mercury, lead, arsenic, cadmium and selenium in infants during early lactation in comparison with placental transfer. Ecotoxicology and environmental safety 84 (2012): 179-84 | Bioavailability not assessed |
| Bioavailability | Gundacker, Claudia; Frohlich, Sonja; Graf-Rohrmeister, Klaudia; Eibenberger, Barbara; Jessenig, Verena; Gicic, Dijana; Prinz, Susanne; Wittmann, Karl Johann; Zeisler, Harald; Vallant, Birgit; Pollak, Arnold; Husslein, Peter. Perinatal lead and mercury exposure in Austria. The Science of the total environment 408 (2010): 5744-9 | Bioavailability not assessed |
| Bioavailability | Fromme, Hermann; Mosch, Christine; Morovitz, Maria; Alba-Alejandre, Irene; Boehmer, Sigrun; Kiranoglu, Mandy; Faber, Fabienne; Hannibal, Iris; Genzel-Boroviczeny, Orsolya; Koletzko, Berthold; Volkel, Wolfgang. Pre- and postnatal exposure to perfluorinated compounds (PFCs). Environmental science & technology 44 (2010): 7123-9 | Study design |
| Bioavailability | Marques, Rejane C; Dorea, Jose G; Bernardi, Jose V E; Bastos, Wanderley R; Malm, Olaf. Prenatal and postnatal mercury exposure, breastfeeding and neurodevelopment during the first 5 years. Cognitive and behavioral neurology: official journal of the Society for Behavioral and Cognitive Neurology 22 (2009): 134-41 | Bioavailability not assessed |
| Bioavailability | Marques, Rejane C; Dorea, Jose G; Bastos, Wanderley R; Malm, Olaf. Changes in children hair-Hg concentrations during the first 5 years: maternal, environmental and iatrogenic modifying factors. Regulatory toxicology and pharmacology: RTP 49 (2007): 17-24 | Bioavailability not assessed |
| Bioavailability | Bjornberg, Karolin Ask; Vahter, Marie; Berglund, Birgitta; Niklasson, Boel; Blennow, Mats; Sandborgh-Englund, Gunilla. Transport of methylmercury and inorganic mercury to the fetus and breast-fed infant. Environmental health perspectives 113 (2005): 1381-5 | Bioavailability not assessed |
| Bioavailability | Hanning, Rhona M; Sandhu, Ranjit; MacMillan, Angus; Moss, Lorraine; Tsuji, Leonard J S; Nieboer, Evert. Impact on blood Pb levels of maternal and early infant feeding practices of First Nation Cree in the Mushkegowuk Territory of northern Ontario, Canada. Journal of environmental monitoring: JEM 5 (2003): 241-5 | Study design |
| Bioavailability | Sakamoto, Mineshi; Kubota, Machi; Matsumoto, Shin-ichiro; Nakano, Atsuhiro; Akagi, Hirokatsu. Declining risk of methylmercury exposure to infants during lactation. Environmental research 90 (2002): 185-9 | Study design |
| Bioavailability | Gulson, B L; Mizon, K J; Palmer, J M; Patison, N; Law, A J; Korsch, M J; Mahaffey, K R; Donnelly, J B. Longitudinal study of daily intake and excretion of lead in newly born infants. Environmental research 85 (2001): 232-45 | Study design |
| Bioavailability | Ramirez, G B; Cruz, M C; Pagulayan, O; Ostrea, E; Dalisay, C. The Tagum study I: analysis and clinical correlates of mercury in maternal and cord blood, breast milk, meconium, and infants' hair. Pediatrics 106 (2000): 774-81 | Bioavailability not assessed |
| Bioavailability | Boischio, A A; Henshel, D S. Linear regression models of methyl mercury exposure during prenatal and early postnatal life among riverside people along the upper Madeira River, Amazon. Environmental research 83 (2000): 150-61 | Bioavailability not assessed |
| Bioavailability | Sargent, J D; Dalton, M A; O'Connor, G T; Olmstead, E M; Klein, R Z. Randomized trial of calcium glycerophosphate-supplemented infant formula to prevent lead absorption. The American journal of clinical nutrition 69 (1999): 1224-30 | Bioavailability not assessed |
| Bioavailability | Gulson, B L; Jameson, C W; Mahaffey, K R; Mizon, K J; Patison, N; Law, A J; Korsch, M J; Salter, M A. Relationships of lead in breast milk to lead in blood, urine, and diet of the infant and mother. Environmental health perspectives 106 (1998): 667-74 | Study design |
| Bioavailability | Concha, G; Vogler, G; Lezcano, D; Nermell, B; Vahter, M. Exposure to inorganic arsenic metabolites during early human development. Toxicological sciences: an official journal of the Society of Toxicology 44 (1998): 185-90 | Bioavailability not assessed |
| Bioavailability | Concha, G; Vogler, G; Nermell, B; Vahter, M. Low-level arsenic excretion in breast milk of native Andean women exposed to high levels of arsenic in the drinking water. International archives of occupational and environmental health 71 (1998): 42-6 | Study design |
| Bioavailability | Skerfving, S. Mercury in women exposed to methylmercury through fish consumption, and in their newborn babies and breast milk. Bulletin of environmental contamination and toxicology 41 (1988): 475-82 | Study design |
| Bioavailability | Sherlock, J C; Quinn, M J. Relationship between blood lead concentrations and dietary lead intake in infants: the Glasgow Duplicate Diet Study 1979-1980. Food additives and contaminants 3 (1986): 167-76 | Bioavailability not assessed |
| Bioavailability | Rabinowitz, M; Leviton, A; Needleman, H. Lead in milk and infant blood: a dose-response model. Archives of environmental health 40 (1985): 283-6 | Bioavailability not assessed |
| Bioavailability | Kovar, I Z; Strehlow, C D; Richmond, J; Thompson, M G. Perinatal lead and cadmium burden in a British urban population. Archives of disease in childhood 59 (1984): 36-9 | Bioavailability not assessed |
| Bioavailability | Rye, J E; Ziegler, E E; Nelson, S E; Fomon, S J. Dietary intake of lead and blood lead concentration in early infancy. American journal of diseases of children (1960) 137 (1983): 886-91 | Study design |
| Bioavailability | Fujita, M; Takabatake, E. Mercury levels in human maternal and neonatal blood, hair and milk. Bulletin of environmental contamination and toxicology 18 (1977): 205-9 | Bioavailability not assessed |
| Bioavailability | Galster, W A. Mercury in Alaskan Eskimo mothers and infants. Environmental health perspectives 15 (1976): 135-40 | Bioavailability not assessed |
| Bioavailability | Mansour S.A.A.; Mohmoud A.M.A.; Ibrahim H.I.M.; Abdelghaffar M.A.S.; Marai A.A.M.; Abdul-Aziz A.E.; Abdrabo A.E.M. Mercury and Lead Levels in Pregnant Women and Their Newborns After Delivery in Cairo, Egypt. NeuroQuantology 20 (2022): 884-891 | Bioavailability not assessed |
| Bioavailability | Ernawati; Sutomo A.H.; Indwiani A.A. Overview of 8-hydroxy-2'-deoxyguanosine (8-ohdg) as DNA damage biomarker in infants who live near gold mines, Yogyakarta, Indonesia. Indian Journal of Public Health Research and Development 12 (2021): 101-105 | Study design |
| Bioavailability | Plockinger B.; Ulm M.R.; Golaszewski T.; Meisinger V.; Suzin J.; Grudzinska M.; Zdziennicki A.; Dadak C. Lead, mercury, and cadmium exposure of neonates in Poland compared to Austria and other European countries. Trace Elements and Electrocytes 13 (1996): 22-25 | Study design |
| Bioavailability | Hamzaoglu, Onur; Yavuz, Melike; Turker, Gulcan; Savli, Hakan. Air Pollution and Heavy Metal Concentration in Colostrum and Meconium in Two Different Districts of an Industrial City: A Preliminary Report. International Medical Journal 21 (2014): 77-82 | Publication type |
| Bioavailability | Trdin, A.; Snoj Tratnik, J.; Stajnko, A.; Marc, J.; Mazej, D.; Sesek Briski, A.; Kastelec, D.; Prpic, I.; Petrovic, O.; Spiric, Z.; Horvat, M.; Falnoga, I. Trace elements and APOE polymorphisms in pregnant women and their new-borns. Environment International 143 (2020): 105626 | Bioavailability not assessed |
| Bioavailability | Okati, N.; Sari, A. E.; Ghasempouri, S. M. Evaluation of mercury pollution in breast milk and Iranian infants' hair. International Research Journal of Applied and Basic Sciences 4 (2013): 2857-2864 | Bioavailability not assessed |
| Bioavailability | Tatsuta, N.; Asato, K.; Iwai-Shimada, M.; Iwai, K.; Nakayama, S.F.; Yamazaki, S.; Nakai, K. Dietary intake of methylmercury by 0-5 years children using the duplicate diet method in Japan. Environmental Health and Preventive Medicine 29 (2024): 27 | Bioavailability not assessed |

^a^Full texts that were identified from the backwards citation search of the reference lists of included articles in the synthesis.

^b^Full texts that were identified from the manual search of the *Journal of Environmental Exposure Assessment*

## **Supplemental Table 9**: Excluded full text articles identified from the HOME Study website, HOME study trial registry (NCT), or Rochester Lead-in-Dust study website* with reasons for exclusion.

| **Source** | **Article** | **Reason for exclusion** |
| --- | --- | --- |
| HOME study | Hall, AM; Ashley-Martin, J; Lei Liang, C; Papandonatos, GD; Arbuckle, TE; Borghese, MM; Buckley, JP; Cecil, KM; Chen, A; Dodds, L; Fisher, M; Lanphear, BP; Fk Rawn, D; Yolton, K; Braun, JM. Personal care product use and per- and polyfluoroalkyl substances in pregnant and lactating people in the Maternal-Infant Research on Environmental Chemicals study. Environment International. 2024; 193:109094. | Duplicate record with database search |
| HOME study | Braun, JM; Papandonatos, GD; Li, N; Sears, CG; Buckley, JP; Cecil, KM; Chen, A; Eaton, CB; Kalkwarf, HJ; Kelsey, KT; Lanphear, BP; Yolton, K. Physical activity modifies the relation between gestational perfluorooctanoic acid exposure and adolescent cardiometabolic risk. Environmental Research. 2022; 214(Pt 3):114021. | Duplicate record with database search |
| HOME study | Fleury, ES; Kuiper, JR; Buckley, JP; Papandonatos, GD; Cecil, KM; Chen, A; Eaton, CB; Kalkwarf, HJ; Lanphear, BP; Yolton, K; Braun, JM. Evaluating the association between longitudinal exposure to a PFAS mixture and adolescent cardiometabolic risk in the HOME Study. Environmental Epidemiology. 2024; 8(1):e289. | Outcome  Cord blood (ineligible as an infant biomarker because exposure to human milk or infant formula did not precede biospecimen collection). |
| HOME study | Fossa, AJ; Manz, KE; Papandonatos, GD; Chen, A; La Guardia, MJ; Lanphear, BP; C Hale, R; Pagano, A; Pennell, KD; Yolton, K; Braun, JM. A randomized controlled trial of a housing intervention to reduce endocrine disrupting chemical exposures in children. Environment International. 2024; 191:108994. | Outcome  Cord blood (ineligible as an infant biomarker because exposure to human milk or infant formula did not precede biospecimen collection). |
| HOME study | Kuiper, JR; Liu, SH; Lanphear, BP; Calafat, AM; Cecil, KM; Xu, Y; Yolton, K; Kalkwarf, HJ; Chen, A; Braun, JM; Buckley, JP. Estimating effects of longitudinal and cumulative exposure to PFAS mixtures on early adolescent body composition. American Journal of Epidemiology. 2024; 193(6):917-925. | Outcome  Cord blood (ineligible as an infant biomarker because exposure to human milk or infant formula did not precede biospecimen collection). |
| HOME study | Vasil, T-M; Fleury, ES; Walker, ED; Kuiper, JR; Buckley, JP; Cecil, KM; Chen, A; Kalkwarf, HJ; Lanphear, BP; Yolton, K; Braun, JM. Associations of pre- and postnatal per- and polyfluoroalkyl substance exposure with adolescents' eating behaviors. Environmental Epidemiology. 2024; 8(5):e343. | Outcome  Cord blood (ineligible as an infant biomarker because exposure to human milk or infant formula did not precede biospecimen collection). |
| HOME study | Folger, AT; Ding, L; Yolton, K; Ammerman, RT; Ji, H; Frey, JR; Bowers, KA. Association between maternal prenatal depressive symptoms and offspring epigenetic aging at 3-5 weeks. Annals of Epidemiology. 2024; 93:1-6. | Outcome  Epigenetic age |
| HOME study | Iyanna, N; Yolton, K; Lemasters, G; Lanphear, BP; Cecil, KM; Schwartz, J; Brokamp, C; Rasnick, E; Xu, Y; Macdougall, MC; Ryan, PH. Air pollution exposure and social responsiveness in childhood: The cincinnati combined childhood cohorts. International Journal of Hygiene and Environmental Health. 2023; 251:114172. | Outcome  Neurobehavioral outcomes |
| HOME study | Jia, W; Chen, A; Yolton, K; Xu, Y; Lili, H; Jandarov, RA. Assessing the longitudinal impact of environmental chemical mixtures on children’s neurodevelopment: A Bayesian approach. Hygiene and Environmental Health Advances. 2025; 16:100146. | Outcome  Neurodevelopment outcomes |
| HOME study | Signes-Pastor, AJ; Romano, ME; Jackson, B; Braun, JM; Yolton, K; Chen, A; Lanphear, B; Karagas, MR. Associations of maternal urinary arsenic concentrations during pregnancy with childhood cognitive abilities: The HOME study. International Journal of Hygiene and Environmental Health. 2022; 245:114009. | Population  Arsenic measured in maternal biospecimen only. |
| HOME study | Sears, CG; Lanphear, BP; Xu, Y; Chen, A; Yolton, K; Braun, JM. Identifying periods of heightened susceptibility to lead exposure in relation to behavioral problems. Journal of Exposure Science and Environmental Epidemiology. 2022; 32(1):1-9. | Population  Child blood collected at 1.1 y. Therefore, the measurement occurred after the exposure window of interest (≤ 12 months). Also, data or analyses not reported based on infant feeding (HM only, IF only, or HM and IF). |
| HOME study | Vester, A; Xu, Y; Newman, NC; Macdougall, MC; Papandonatos, GD; Parsons, PJ; Palmer, CD; Braun, JM; Lanphear, BP; Chen, A; Cecil, KM; Yolton, K. Cumulative childhood lead exposure estimation and school-age IQ in a prospective birth cohort. Environmental Health: A Global Access Science Source. 2025; 25(1):1. | Population  Child blood collected at 1.1 y. Therefore, the measurement occurred after the exposure window of interest (≤ 12 months). Also, data or analyses not reported based on infant feeding (HM only, IF only, or HM and IF). |
| HOME study | Braun, JM; Yolton, K; Newman, N; Jacobs, DE; Taylor, M; Lanphear, BP. Residential dust lead levels and the risk of childhood lead poisoning in United States children. Pediatric Research. 2021; 90(4):896-902. | Population  Child blood collected at 1.1 y. Therefore, the measurement occurred after the exposure window of interest (≤ 12 months). Also, data or analyses not reported based on infant feeding (HM only, IF only, or HM and IF). |
| HOME study | Liu, SH; Chen, Y; Feuerstahler, L; Chen, A; Starling, A; Dabelea, D; Wang, X; Cecil, K; Lanphear, B; Yolton, K; Braun, JM; Buckley, JP. The U.S. PFAS exposure burden calculator for 2017-2018: Application to the HOME Study, with comparison of epidemiological findings from NHANES. Neurotoxicology and Teratology. 2024; 102:107321. | Population  Child serum collected at age 12 y. Therefore, the measurement occurred after the exposure window of interest (≤ 12 months). |
| HOME study | Liu, Y; Gairola, R; Kuiper, JR; Papandonatos, GD; Kelsey, KT; Langevin, SM; Buckley, JP; Chen, A; Lanphear, BP; Cecil, KM; Yolton, K; Braun, JM. Lifetime Postnatal Exposure to Perfluoroalkyl Substance Mixture and DNA Methylation at Twelve Years of Age. Environmental Science and Technology Letters. 2023; 10(10):824-830. | Population  Child serum collected at ages 3, 8, and 12 y. Therefore, the measurement occurred after the exposure window of interest (≤ 12 months ). |
| HOME study | Lee, H; Hall, AM; Calafat, AM; Chen, A; Fazili, Z; Lanphear, BP; Pfeiffer, CM; Yolton, K; Braun, JM. Associations of prenatal per- and polyfluoroalkyl substances with whole blood folate levels in pregnant women in the Health Outcomes and Measures of the Environment (HOME) Study. Environmental Epidemiology. 2025; 9(4):e406. | Population  PFAS in maternal blood/serum only. |
| HOME study | Hall, AM; Fleury, E; Papandonatos, GD; Buckley, JP; Cecil, KM; Chen, A; Lanphear, BP; Yolton, K; Walker, DI; Pennell, KD; Braun, JM; Manz, KE. Associations of a Prenatal Serum Per- and Polyfluoroalkyl Substance Mixture with the Cord Serum Metabolome in the HOME Study. Environmental Science and Technology. 2023; 57(51):21627-21636. | Population  PFAS in maternal serum only. |
| HOME study | Buckley, JP; Zhou, J; Marquess, KM; Lanphear, BP; Cecil, KM; Chen, A; Sears, CG; Xu, Y; Yolton, K; Kalkwarf, HJ; Braun, JM; Kuiper, JR. Per- and polyfluoroalkyl substances and bone mineral content in early adolescence: Modification by diet and physical activity. Environmental Research. 2024; 252(Pt 1):118872. | Population  PFAS measured in children at age 12 y. Therefore, the measurement occurred after the exposure window of interest (≤ 12 months). |
| HOME study | Sultan, H; Buckley, JP; Kalkwarf, HJ; Cecil, KM; Chen, A; Lanphear, BP; Yolton, K; Braun, JM. Dietary per- and polyfluoroalkyl substance (PFAS) exposure in adolescents: The HOME study. Environmental Research. 2023; 231(Pt 1):115953. | Population  PFAS measured in children at age 12 y. Therefore, the measurement occurred after the exposure window of interest (≤ 12 months). |
| HOME study | Vuong, AM; Yolton, K; Xie, C; Dietrich, KN; Braun, JM; Webster, GM; Calafat, AM; Lanphear, BP; Chen, A. Childhood exposure to per- and polyfluoroalkyl substances (PFAS) and neurobehavioral domains in children at age 8 years. Neurotoxicology and Teratology. 2021; 88:107022. | Population  PFAS measured in children at ages 3 and 8 y. Therefore, the measurement occurred after the exposure window of interest (≤ 12 months). |
| HOME study | Sears, CG; Liu, Y; Lanphear, BP; Buckley, JP; Meyer, J; Xu, Y; Chen, A; Yolton, K; Braun, JM. Evaluating Mixtures of Urinary Phthalate Metabolites and Serum Per-/Polyfluoroalkyl Substances in Relation to Adolescent Hair Cortisol: The HOME Study. American Journal of Epidemiology. 2024; 193(3):454-468. | Population  PFAS measured in children at ages 3, 8, and 12 y. Therefore, the measurement occurred after the exposure window of interest (≤ 12 months). |
| HOME study | Liu, Y; Eliot, MN; Papandonatos, GD; Kelsey, KT; Fore, R; Langevin, S; Buckley, J; Chen, A; Lanphear, BP; Cecil, KM; Sagiv, SK; Baccarelli, AA; Oken, E; Braun, JM. Gestational Perfluoroalkyl Substance Exposure and DNA Methylation at Birth and 12 Years of Age: A Longitudinal Epigenome-Wide Association Study. Environmental Health Perspectives. 2022; 130(3):37005. | Population  PFAS measured in maternal samples and in children at age 12 y. Therefore, the measurement occurred after the exposure window of interest (≤ 12 months). |
| HOME study | Liu, Y; Calafat, AM; Chen, A; Lanphear, BP; Jones, N-HY; Cecil, KM; Rose, SR; Yolton, K; Buckley, JP; Braun, JM. Associations of prenatal and postnatal exposure to perfluoroalkyl substances with pubertal development and reproductive hormones in females and males: The HOME study. Science of the Total Environment. 2023; 890:164353. | Population  PFAS measured in maternal samples and in children at ages 3, 8, and 12 y. Therefore, the measurement occurred after the exposure window of interest (≤ 12 months). |
| HOME study | Puvvula, J; Hwang, W-T; Mccandless, L; Xie, C; Braun, JM; Vuong, AM; Oulhote, Y; Schisterman, EF; Shinohara, RT; Booij, L; Ashley-Martin, J; Arbuckle, TE; Lanphear, B; Chen, A. Gestational exposure to environmental chemical mixtures and cognitive abilities in children: A pooled analysis of two North American birth cohorts. Environment International. 2025; 196:109298. | Population  PFAS measured in maternal samples only. |
| HOME NCT | Kato K, Wong LY, Chen A, Dunbar C, Webster GM, Lanphear BP, Calafat AM. Changes in serum concentrations of maternal poly- and perfluoroalkyl substances over the course of pregnancy and predictors of exposure in a multiethnic cohort of Cincinnati, Ohio pregnant women during 2003-2006. Environ Sci Technol. 2014 Aug 19;48(16):9600-8. doi: 10.1021/es501811k. Epub 2014 Jul 29. | Duplicate record with database search |
| HOME NCT | Kingsley SL, Eliot MN, Kelsey KT, Calafat AM, Ehrlich S, Lanphear BP, Chen A, Braun JM. Variability and predictors of serum perfluoroalkyl substance concentrations during pregnancy and early childhood. Environ Res. 2018 Aug;165:247-257. doi: 10.1016/j.envres.2018.04.033. Epub 2018 May 7. | Duplicate record with database search |
| HOME NCT | Romano ME, Xu Y, Calafat AM, Yolton K, Chen A, Webster GM, Eliot MN, Howard CR, Lanphear BP, Braun JM. Maternal serum perfluoroalkyl substances during pregnancy and duration of breastfeeding. Environ Res. 2016 Aug;149:239-246. doi: 10.1016/j.envres.2016.04.034. Epub 2016 May 11. | Duplicate record with database search |
| HOME NCT | Rosen-Carole CB, Auinger P, Howard CR, Brownell EA, Lanphear BP. Low-Level Prenatal Toxin Exposures and Breastfeeding Duration: A Prospective Cohort Study. Matern Child Health J. 2017 Dec;21(12):2245-2255. doi: 10.1007/s10995-017-2346-4. | Duplicate record with database search |
| HOME NCT | Spanier AJ, Wilson S, Ho M, Hornung R, Lanphear BP. The contribution of housing renovation to children's blood lead levels: a cohort study. Environ Health. 2013 Aug 27;12:72. doi: 10.1186/1476-069X-12-72. | Outcome  Data or analyses not reported based on infant feeding (HM only, IF only, or HM and IF). |
| HOME NCT | Xu Y, Khoury JC, Sucharew H, Dietrich K, Yolton K. Low-level gestational exposure to mercury and maternal fish consumption: Associations with neurobehavior in early infancy. Neurotoxicol Teratol. 2016 Mar-Apr;54:61-7. doi: 10.1016/j.ntt.2016.02.002. Epub 2016 Feb 12. | Outcome  Cord blood (ineligible as an infant biomarker because exposure to human milk or infant formula did not precede biospecimen measurement). |
| HOME NCT | Patel NB, Xu Y, McCandless LC, Chen A, Yolton K, Braun J, Jones RL, Dietrich KN, Lanphear BP. Very low-level prenatal mercury exposure and behaviors in children: the HOME Study. Environ Health. 2019 Jan 9;18(1):4. doi: 10.1186/s12940-018-0443-5. | Outcome  Cord blood (ineligible as an infant biomarker because exposure to human milk or infant formula did not precede biospecimen measurement). |
| HOME NCT | Braun JM, Kalkwarf HJ, Papandonatos GD, Chen A, Lanphear BP. Patterns of early life body mass index and childhood overweight and obesity status at eight years of age. BMC Pediatr. 2018 May 11;18(1):161. doi: 10.1186/s12887-018-1124-9. | Outcome  Anthropometric measurements. |
| HOME NCT | Braun JM, Daniels JL, Poole C, Olshan AF, Hornung R, Bernert JT, Khoury J, Needham LL, Barr DB, Lanphear BP. Prenatal environmental tobacco smoke exposure and early childhood body mass index. Paediatr Perinat Epidemiol. 2010 Nov;24(6):524-34. doi: 10.1111/j.1365-3016.2010.01146.x. Epub 2010 Aug 16. | Outcome  Anthropometric measurements. |
| HOME NCT | Braun JM, Hornung R, Chen A, Dietrich KN, Jacobs DE, Jones R, Khoury JC, Liddy-Hicks S, Morgan S, Vanderbeek SB, Xu Y, Yolton K, Lanphear BP. Effect of Residential Lead-Hazard Interventions on Childhood Blood Lead Concentrations and Neurobehavioral Outcomes: A Randomized Clinical Trial. JAMA Pediatr. 2018 Oct 1;172(10):934-942. doi: 10.1001/jamapediatrics.2018.2382. | Population  Child blood collected at 1.1 y. Therefore, the measurement occurred after the exposure window of interest (≤ 12 months). Also, data or analyses not reported based on infant feeding (HM only, IF only, or HM and IF). |
| HOME NCT | Braun JM, Kalloo G, Chen A, Dietrich KN, Liddy-Hicks S, Morgan S, Xu Y, Yolton K, Lanphear BP. Cohort Profile: The Health Outcomes and Measures of the Environment (HOME) study. Int J Epidemiol. 2017 Feb 1;46(1):24. doi: 10.1093/ije/dyw006. No abstract available. | Population  Cohort profile. Child blood collected at 1.1 y. Therefore, the measurement occurred after the exposure window of interest (≤ 12 months). |
| HOME NCT | Woods MM, Lanphear BP, Braun JM, McCandless LC. Gestational exposure to endocrine disrupting chemicals in relation to infant birth weight: a Bayesian analysis of the HOME Study. Environ Health. 2017 Oct 27;16(1):115. doi: 10.1186/s12940-017-0332-3. | Population  Contaminants measured in maternal samples (urine, blood) only. |
| HOME NCT | Lanphear BP, Hornung R, Khoury J, Yolton K, Baghurst P, Bellinger DC, Canfield RL, Dietrich KN, Bornschein R, Greene T, Rothenberg SJ, Needleman HL, Schnaas L, Wasserman G, Graziano J, Roberts R. Low-level environmental lead exposure and children's intellectual function: an international pooled analysis. Environ Health Perspect. 2005 Jul;113(7):894-9. doi: 10.1289/ehp.7688. | Population  Mean blood lead reported from 6 to 24 months. Therefore, the mean measurement occurred after the exposure window of interest (≤ 12 months). Also, data or analyses not reported based on infant feeding (HM only, IF only, or HM and IF). |
| HOME NCT | Dasu K, Nakayama SF, Yoshikane M, Mills MA, Wright JM, Ehrlich S. An ultra-sensitive method for the analysis of perfluorinated alkyl acids in drinking water using a column switching high-performance liquid chromatography tandem mass spectrometry. J Chromatogr A. 2017 Apr 21;1494:46-54. doi: 10.1016/j.chroma.2017.03.006. Epub 2017 Mar 6. | Population  Non-human study. PFAS measurement in water only. |
| HOME NCT | Kalloo G, Wellenius GA, McCandless L, Calafat AM, Sjodin A, Karagas M, Chen A, Yolton K, Lanphear BP, Braun JM. Profiles and Predictors of Environmental Chemical Mixture Exposure among Pregnant Women: The Health Outcomes and Measures of the Environment Study. Environ Sci Technol. 2018 Sep 4;52(17):10104-10113. doi: 10.1021/acs.est.8b02946. Epub 2018 Aug 21. | Population  PFAS and heavy metals in maternal biospecimens (urine, serum, and blood) only. |
| HOME NCT | Buck CO, Eliot MN, Kelsey KT, Calafat AM, Chen A, Ehrlich S, Lanphear BP, Braun JM. Prenatal exposure to perfluoroalkyl substances and adipocytokines: the HOME Study. Pediatr Res. 2018 Dec;84(6):854-860. doi: 10.1038/s41390-018-0170-1. Epub 2018 Sep 13. | Population  PFAS in maternal serum only. |
| HOME NCT | Donauer S, Chen A, Xu Y, Calafat AM, Sjodin A, Yolton K. Prenatal exposure to polybrominated diphenyl ethers and polyfluoroalkyl chemicals and infant neurobehavior. J Pediatr. 2015 Mar;166(3):736-42. doi: 10.1016/j.jpeds.2014.11.021. Epub 2014 Dec 16. | Population  PFAS in maternal serum only. |
| HOME NCT | Vuong AM, Braun JM, Yolton K, Wang Z, Xie C, Webster GM, Ye X, Calafat AM, Dietrich KN, Lanphear BP, Chen A. Prenatal and childhood exposure to perfluoroalkyl substances (PFAS) and measures of attention, impulse control, and visual spatial abilities. Environ Int. 2018 Oct;119:413-420. doi: 10.1016/j.envint.2018.07.013. Epub 2018 Jul 20. | Population  PFAS measured in children at ages 3 and 8 y. Therefore, the measurement occurred after the exposure window of interest (≤ 12 months). |
| HOME NCT | Zhang H, Yolton K, Webster GM, Ye X, Calafat AM, Dietrich KN, Xu Y, Xie C, Braun JM, Lanphear BP, Chen A. Prenatal and childhood perfluoroalkyl substances exposures and children's reading skills at ages 5 and 8years. Environ Int. 2018 Feb;111:224-231. doi: 10.1016/j.envint.2017.11.031. Epub 2017 Dec 20. | Population  PFAS measured in children at ages 3 and 8 y. Therefore, the measurement occurred after the exposure window of interest (≤ 12 months). |
| HOME NCT | Braun JM, Kalkbrenner AE, Just AC, Yolton K, Calafat AM, Sjodin A, Hauser R, Webster GM, Chen A, Lanphear BP. Gestational exposure to endocrine-disrupting chemicals and reciprocal social, repetitive, and stereotypic behaviors in 4- and 5-year-old children: the HOME study. Environ Health Perspect. 2014 May;122(5):513-20. doi: 10.1289/ehp.1307261. Epub 2014 Mar 12. | Population  PFAS measured in maternal samples only. |
| HOME NCT | Braun JM, Chen A, Romano ME, Calafat AM, Webster GM, Yolton K, Lanphear BP. Prenatal perfluoroalkyl substance exposure and child adiposity at 8 years of age: The HOME study. Obesity (Silver Spring). 2016 Jan;24(1):231-7. doi: 10.1002/oby.21258. Epub 2015 Nov 11. | Population  PFAS measured in maternal serum only. |
| HOME NCT | Braun JM, Buckley JP, Cecil KM, Chen A, Kalkwarf HJ, Lanphear BP, Xu Y, Woeste A, Yolton K. Adolescent follow-up in the Health Outcomes and Measures of the Environment (HOME) Study: cohort profile. BMJ Open. 2020 May 7;10(5):e034838. doi: 10.1136/bmjopen-2019-034838. | Publication type  Protocol/description of new wave of data for 12 y old adolescents. |
| Rochester Lead-in-Dust study | University of Rochester School of Medicine Departments of Pediatrics, Biostatistics, and Environmental Medicine, & National Center for Lead-Safe Housing. (1995, June). The relation of lead-contaminated house dust and blood lead levels among urban children: Final report volume II: Results and discussion. Columbia, MD: National Center for Lead-Safe Housing. | Population  Children were aged 12-31 months old. Therefore, the measurement occurred after the exposure window of interest (≤ 12 months). |
| Rochester Lead-in-Dust study | University of Rochester School of Medicine Departments of Pediatrics, Biostatistics, and Environmental Medicine, & National Center for Lead-Safe Housing. (1995, June). The relation of lead-contaminated house dust and blood lead levels among urban children: Final report volume I: Protocols and forms. Columbia, MD: National Center for Lead-Safe Housing. | Population  Children were aged 12-31 months old. Therefore, the measurement occurred after the exposure window of interest (≤ 12 months). |
| Rochester Lead-in-Dust study | Lanphear, B. P., Weitzman, M., Winter, N. L., Eberly. S. Yakir, B., Tanner, M., Emond, M., et al (1996, October). Lead-contaminated house dust and urban children’s blood lead levels. American Journal of Public Health, 86(10), 1416-1421. | Population  Children were aged 12-31 months old. Therefore, the measurement occurred after the exposure window of interest (≤ 12 months). |
| Rochester Lead-in-Dust study | Lanphear, B. P., Weitzman, M., & Eberly. S. (1996, October). Racial differences in urban children’s environmental exposures to lead. American Journal of Public Health, 86(10), 1460-1463. | Population  Children were aged 12-31 months old. Therefore, the measurement occurred after the exposure window of interest (≤ 12 months). |
| Rochester Lead-in-Dust study | Emond, M. J., Lanphear, B. P., Watts, A., & Eberly, S. (1997, January). Measurement error and its impact on the estimated relationship between dust lead and children’s blood lead: Members of the Rochester Lead-in-Dust Study Group. Environmental Research, 72(1), 82-92. | Population  Children were aged 12-31 months old. Therefore, the measurement occurred after the exposure window of interest (≤ 12 months). |
| Rochester Lead-in-Dust study | Lanphear, B. P., & Roghmann, K. J. (1997). Pathways of lead exposure in urban children. Environmental Research, 74(1), 67-73. | Population  Children were aged 12-31 months old. Therefore, the measurement occurred after the exposure window of interest (≤ 12 months). |
| Rochester Lead-in-Dust study | Rust, S. W., Burgoon, D. A., Lanphear, B. P., & Eberly, S. (1997, February). Log-additive versus log-linear analysis of lead-contaminated house dust and children’s blood-lead levels. Implications for residential dust-lead standards. Environmental Research, 72(2), 173-184. | Population  Children were aged 12-31 months old. Therefore, the measurement occurred after the exposure window of interest (≤ 12 months). |
| Rochester Lead-in-Dust study | Lanphear, B. P., Burgoon, D. A., Rust, S. W., Eberly, S., & Galke, W. (1998, February). Environmental exposures to lead and urban children’s blood lead levels. Environmental Research, 76(2), 120-130. | Population  Children were aged 12-31 months old. Therefore, the measurement occurred after the exposure window of interest (≤ 12 months). |

*A clinical trial registry for the Rochester Lead-in-Dust study was not found, likely because this study was conducted prior to the development of clinicaltrials.gov. HM, human milk; IF, infant formula.

## **Supplemental Table 10**: Measured median (min-max) urinary arsenic species reported in infants by feeding practice (Signes-Pastor, 2018)

| **Inorganic arsenic (µg/L)** | **MMA (µg/L)** | **DMA (µg/L)** | **Arsenobetaine (µg/L)** |
| --- | --- | --- | --- |
| **Before weaning (4 months old)** | | | |
| All infants (n=15): 0.109 (0.051 − 0.295)  Exclusively fed HM (n=11): 0.101 (0.051 − 0.162)  Exclusively fed formula (n=1): 0.151 (0.151 − 0.151)  Combination fed (n=3): 0.178 (0.068 − 0.295) | All infants (n=15): 0.019 (<LOD − 0.188)  Exclusively fed HM (n=11): 0.015 (<LOD − 0.021)  Exclusively fed formula (n=1): 0.188 (0.188 − 0.188)  Combination fed (n=3): 0.164 (0.020 − 0.180) | All infants (n=15): 0.131 (0.032 − 1.202)  Exclusively fed HM (n=11): 0.126 (0.032 − 1.202)  Exclusively fed formula (n=1): 1.157 (1.157 − 1.157)  Combination fed (n=3): 0.661 (0.100 − 0.908) | All infants (n=15): 0.061 (0.016 − 0.241)  Exclusively fed HM (n=11): 0.057 (0.016 − 0.139)  Exclusively fed formula (n=1): 0.021 (0.021 − 0.021)  Combination fed (n=3): 0.078 (0.075 − 0.241) |
| **During weaning when solid foods were also consumed (6 months old)** | | | |
| All infants (n=15): 0.160 (0.049 − 1.453)  Fed HM, no formula (n=10): 0.157 (0.049 − 0.368)  Fed formula, no HM (n=3): 0.231 (0.145 − 0.527)  Fed formula and HM (n=2): 1.076 (0.699 − 1.453) | All infants (n=15): 0.105 (0.013 − 1.869)  Fed HM, no formula (n=10): 0.053 (0.013 − 0.565)  Fed formula, no HM (n=3): 0.203 (0.134 − 0.565)  Fed formula and HM (n=2): 1.242 (0.614 − 1.869) | All infants (n=15): 0.759 (0.094 − 9.384)  Fed HM, no formula (n=10): 0.367 (0.094 − 4.055)  Fed formula, no HM (n=3): 1.091 (1.005 − 2.179)  Fed formula and HM (n=2): 7.138 (4.891 − 9.384) | All infants (n=15): 0.060 (0.017 − 0.116)  Fed HM, no formula (n=10): 0.058 (0.017 − 0.116)  Fed formula, no HM (n=3): 0.019 (0.017 − 0.116)  Fed formula and HM (n=2): 0.078 (0.061 − 0.096) |
| **Difference between 6 to 4 months** | | | |
| All infants (n=15)  0.081 (-0.113 − 1.399) | All infants (n=15)  0.045 (-0.054 − 1.689) | All infants (n=15)  0.430 (-0.709 − 8.476) | All infants (n=15)  0.005 (-0.222 − 0.052) |

HM, human milk

## **Supplemental Figure 1.** Analytic framework for ‘What are biospecimen concentrations of arsenic, cadmium, lead, mercury, or PFAS of infants living in the United States that are consuming human milk and/or infant formula?’ (PROSPERO: CRD42024530344).


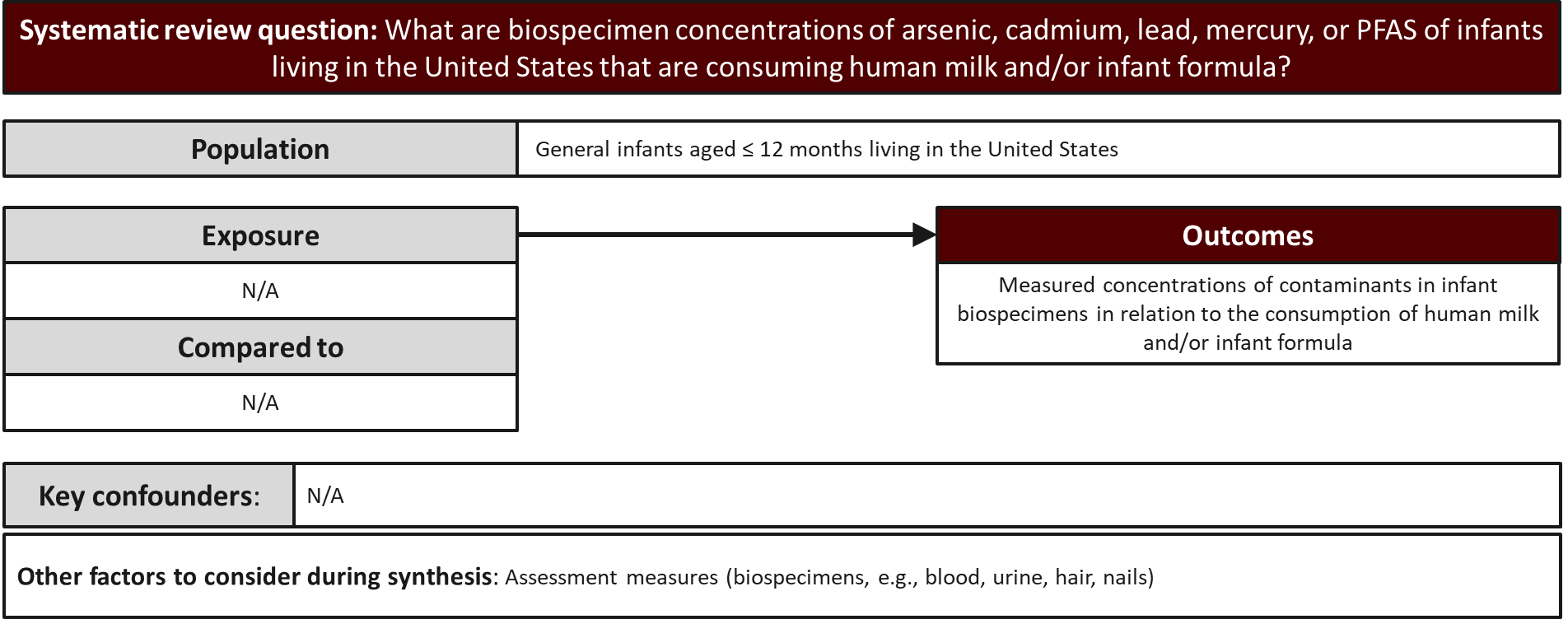


N/A, not applicable.

## **Supplemental Figure 2.** Analytic framework for ‘What is the bioavailability of arsenic, cadmium, lead, mercury, or PFAS from human milk and infant formula when consumed by the infant?’ (PROSPERO: CRD42024530332).


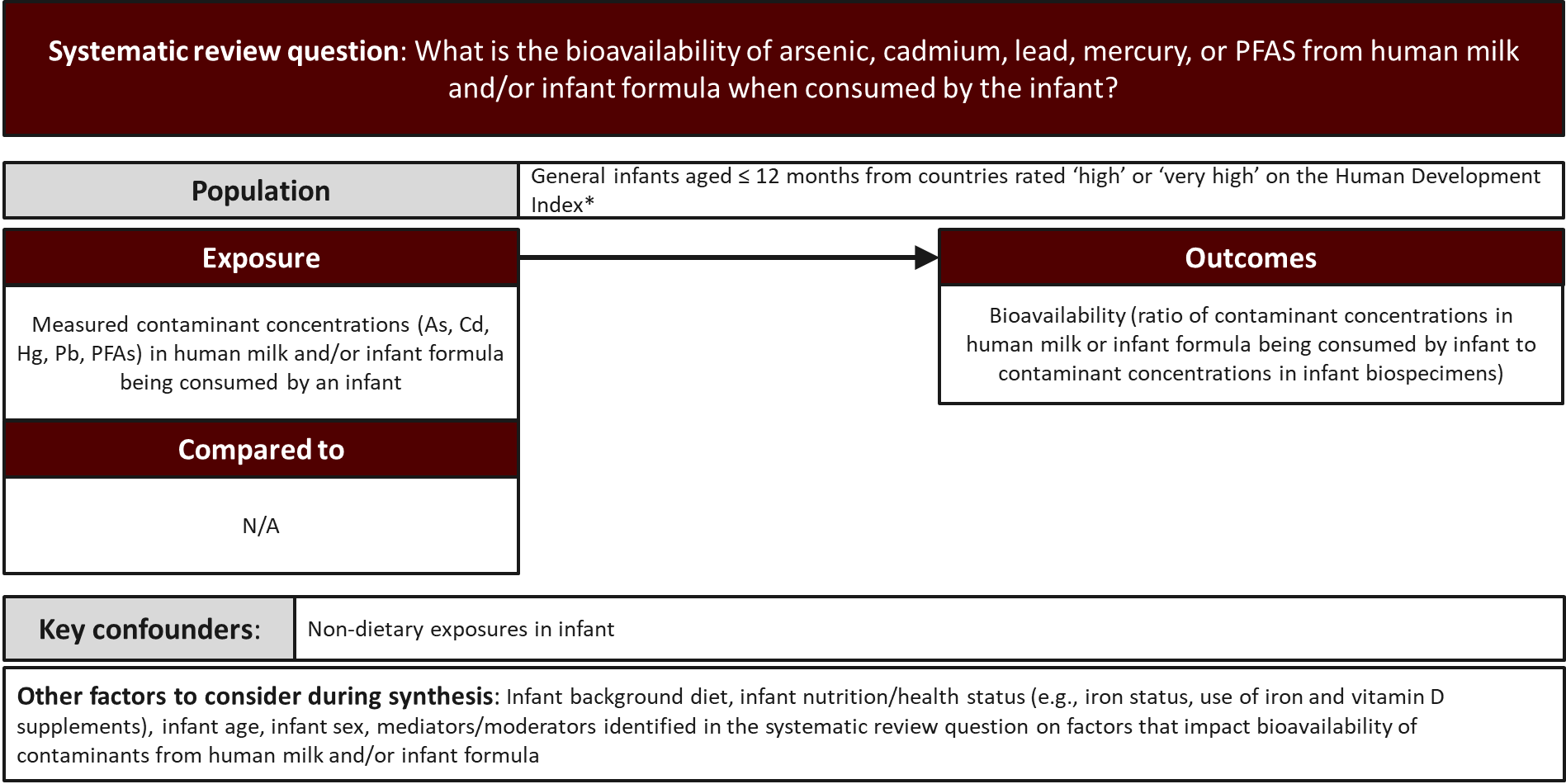


*Based on the Human Development Index which is a “summary measure of average achievement in key dimensions of human development: a long and healthy life, being knowledgeable and having a decent standard of living.”
<https://hdr.undp.org/data-center/human-development-index>
N/A, not applicable.

## **Supplemental Figure 3.** Analytic framework for ‘What factors impact the bioavailability of arsenic, cadmium, lead, mercury, or PFAS from human milk and infant formula when consumed by the infant?’ (PROSPERO: CRD42024530336).


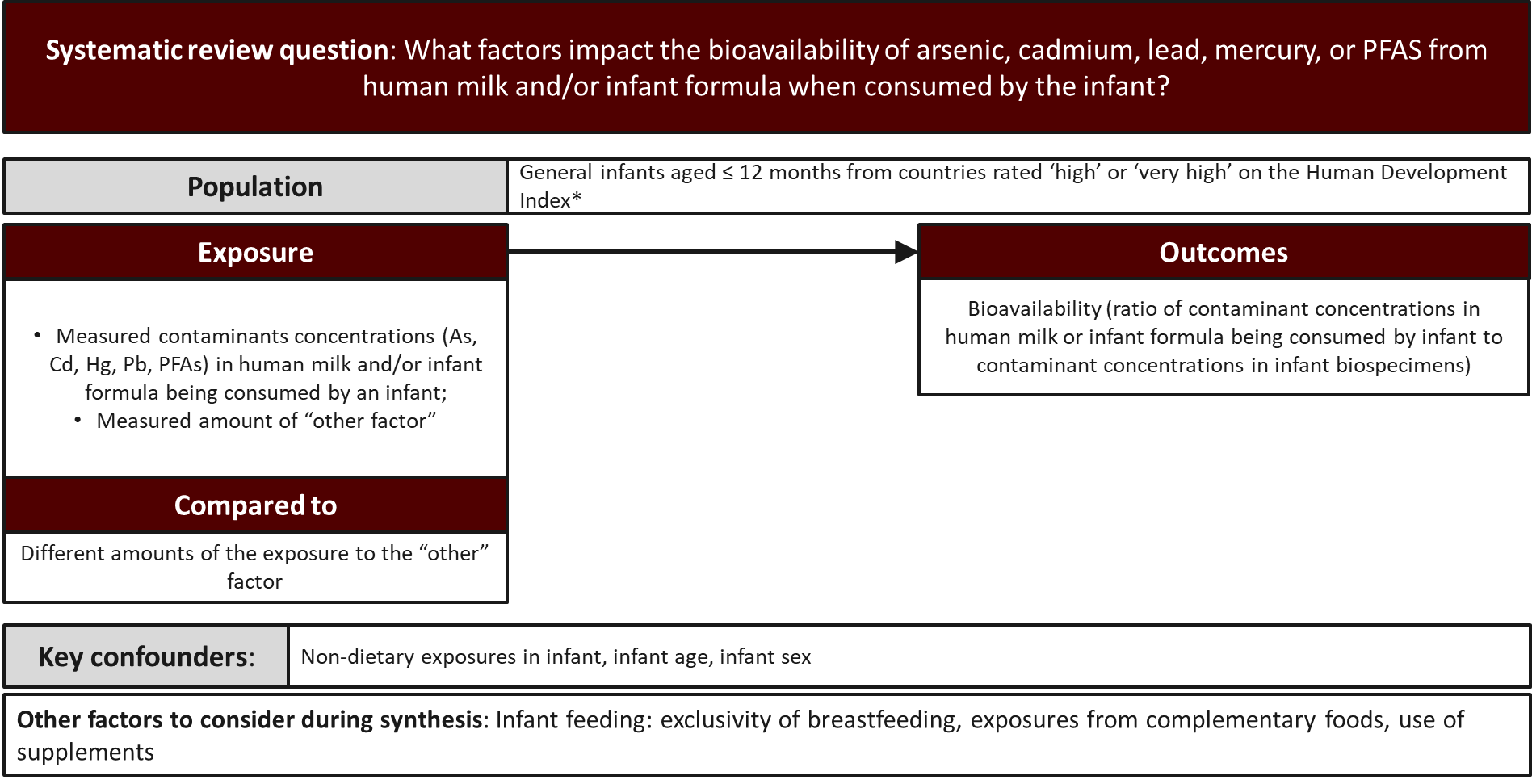


*Based on the Human Development Index which is a “summary measure of average achievement in key dimensions of human development: a long and healthy life, being knowledgeable and having a decent standard of living.”
<https://hdr.undp.org/data-center/human-development-index>

## **Supplemental Figure 4.** Analytic framework for ‘What is the relationship between arsenic, cadmium, lead, mercury, or PFAS and the bioavailability of other components in human milk and infant formula?’ (PROSPERO: CRD42024530339).


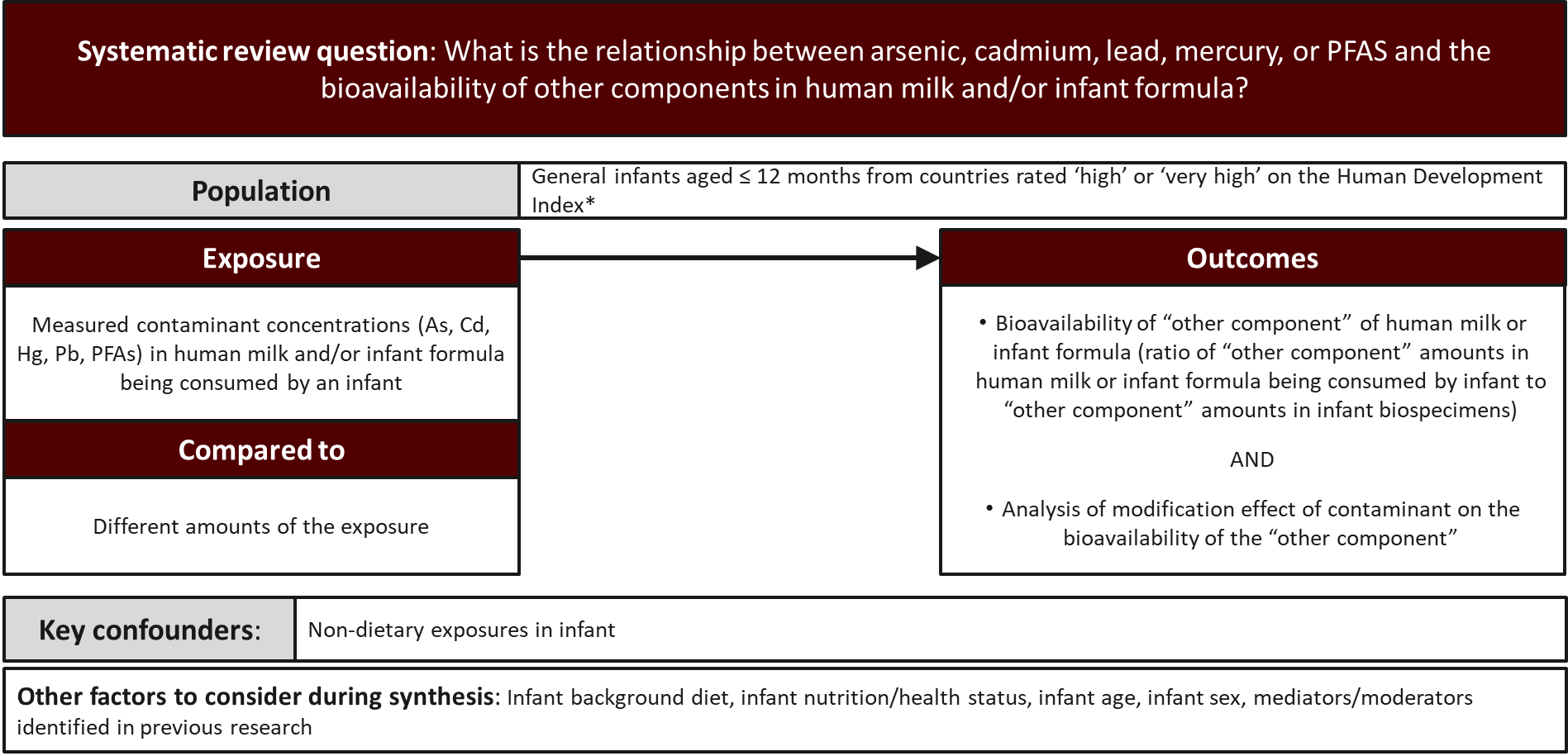


*Based on the Human Development Index which is a “summary measure of average achievement in key dimensions of human development: a long and healthy life, being knowledgeable and having a decent standard of living.”
<https://hdr.undp.org/data-center/human-development-index>

## **Appendix A.** PRISMA 2020 checklist.

| **Section and Topic** | **Item #** | **Checklist item** | **Location where item is reported** |
| --- | --- | --- | --- |
| **TITLE** | | | |
| Title | 1 | Identify the report as a systematic review. | Title Page |
| **ABSTRACT** | | | |
| Abstract | 2 | See the PRISMA 2020 for Abstracts checklist. | Abstract section; Journal word limits precluded our ability to include all inclusion/exclusion criteria and the funding source |
| **INTRODUCTION** | | | |
| Rationale | 3 | Describe the rationale for the review in the context of existing knowledge. | Introduction |
| Objectives | 4 | Provide an explicit statement of the objective(s) or question(s) the review addresses. | Introduction; Figure 1 |
| **METHODS** | | | |
| Eligibility criteria | 5 | Specify the inclusion and exclusion criteria for the review and how studies were grouped for the syntheses. | Supplemental Tables 3-6; Data Synthesis |
| Information sources | 6 | Specify all databases, registers, websites, organisations, reference lists and other sources searched or consulted to identify studies. Specify the date when each source was last searched or consulted. | Supplemental Table 2; Search Strategy |
| Search strategy | 7 | Present the full search strategies for all databases, registers and websites, including any filters and limits used. | Supplemental Table 2 |
| Selection process | 8 | Specify the methods used to decide whether a study met the inclusion criteria of the review, including how many reviewers screened each record and each report retrieved, whether they worked independently, and if applicable, details of automation tools used in the process. | Screening and Data Extraction |
| Data collection process | 9 | Specify the methods used to collect data from reports, including how many reviewers collected data from each report, whether they worked independently, any processes for obtaining or confirming data from study investigators, and if applicable, details of automation tools used in the process. | Screening and Data Extraction |
| Data items | 10a | List and define all outcomes for which data were sought. Specify whether all results that were compatible with each outcome domain in each study were sought (e.g. for all measures, time points, analyses), and if not, the methods used to decide which results to collect. | Screening and Data Extraction |
|  | 10b | List and define all other variables for which data were sought (e.g. participant and intervention characteristics, funding sources). Describe any assumptions made about any missing or unclear information. | Screening and Data Extraction |
| Study risk of bias assessment | 11 | Specify the methods used to assess risk of bias in the included studies, including details of the tool(s) used, how many reviewers assessed each study and whether they worked independently, and if applicable, details of automation tools used in the process. | Risk of Bias |
| Effect measures | 12 | Specify for each outcome the effect measure(s) (e.g. risk ratio, mean difference) used in the synthesis or presentation of results. | N/A |
| Synthesis methods | 13a | Describe the processes used to decide which studies were eligible for each synthesis (e.g. tabulating the study intervention characteristics and comparing against the planned groups for each synthesis (item #5)). | Critical Appraisal of Contaminant Assessment Methods; Data Synthesis |
|  | 13b | Describe any methods required to prepare the data for presentation or synthesis, such as handling of missing summary statistics, or data conversions. | Screening and Data Extraction; |
|  | 13c | Describe any methods used to tabulate or visually display results of individual studies and syntheses. | Data Synthesis |
|  | 13d | Describe any methods used to synthesize results and provide a rationale for the choice(s). If meta-analysis was performed, describe the model(s), method(s) to identify the presence and extent of statistical heterogeneity, and software package(s) used. | Data Synthesis |
|  | 13e | Describe any methods used to explore possible causes of heterogeneity among study results (e.g. subgroup analysis, meta-regression). | Data Synthesis |
|  | 13f | Describe any sensitivity analyses conducted to assess robustness of the synthesized results. | Data Synthesis |
| Reporting bias assessment | 14 | Describe any methods used to assess risk of bias due to missing results in a synthesis (arising from reporting biases). | Risk of Bias |
| Certainty assessment | 15 | Describe any methods used to assess certainty (or confidence) in the body of evidence for an outcome. | Certainty of Evidence |
| **RESULTS** | | | |
| Study selection | 16a | Describe the results of the search and selection process, from the number of records identified in the search to the number of studies included in the review, ideally using a flow diagram. | Search Results; Figures 2 and 4 |
|  | 16b | Cite studies that might appear to meet the inclusion criteria, but which were excluded, and explain why they were excluded. | Supplemental Tables 8 and 9 |
| Study characteristics | 17 | Cite each included study and present its characteristics. | Tables 1-2 |
| Risk of bias in studies | 18 | Present assessments of risk of bias for each included study. | Figure 3 |
| Results of individual studies | 19 | For all outcomes, present, for each study: (a) summary statistics for each group (where appropriate) and (b) an effect estimate and its precision (e.g. confidence/credible interval), ideally using structured tables or plots. | Tables 1-2 |
| Results of syntheses | 20a | For each synthesis, briefly summarise the characteristics and risk of bias among contributing studies. | Throughout results |
|  | 20b | Present results of all statistical syntheses conducted. If meta-analysis was done, present for each the summary estimate and its precision (e.g. confidence/credible interval) and measures of statistical heterogeneity. If comparing groups, describe the direction of the effect. | N/A |
|  | 20c | Present results of all investigations of possible causes of heterogeneity among study results. | Throughout results and discussion |
|  | 20d | Present results of all sensitivity analyses conducted to assess the robustness of the synthesized results. | N/A |
| Reporting biases | 21 | Present assessments of risk of bias due to missing results (arising from reporting biases) for each synthesis assessed. | Results; Figure 3 |
| Certainty of evidence | 22 | Present assessments of certainty (or confidence) in the body of evidence for each outcome assessed. | N/A |
| **DISCUSSION** | | | |
| Discussion | 23a | Provide a general interpretation of the results in the context of other evidence. | Throughout the discussion |
|  | 23b | Discuss any limitations of the evidence included in the review. | Throughout the discussion |
|  | 23c | Discuss any limitations of the review processes used. | Last paragraph of discussion |
|  | 23d | Discuss implications of the results for practice, policy, and future research. | Throughout the discussion |
| **OTHER INFORMATION** | | | |
| Registration and protocol | 24a | Provide registration information for the review, including register name and registration number, or state that the review was not registered. | Methods; Figure 1 |
|  | 24b | Indicate where the review protocol can be accessed, or state that a protocol was not prepared. | Methods |
|  | 24c | Describe and explain any amendments to information provided at registration or in the protocol. | Methods |
| Support | 25 | Describe sources of financial or non-financial support for the review, and the role of the funders or sponsors in the review. | Acknowledgements |
| Competing interests | 26 | Declare any competing interests of review authors. | Title page |
| Availability of data, code and other materials | 27 | Report which of the following are publicly available and where they can be found: template data collection forms; data extracted from included studies; data used for all analyses; analytic code; any other materials used in the review. | Data Synthesis |

*From:*  Page MJ, McKenzie JE, Bossuyt PM, Boutron I, Hoffmann TC, Mulrow CD, et al. The PRISMA 2020 statement: an updated guideline for reporting systematic reviews. BMJ 2021;372:n71. doi: 10.1136/bmj.n71. This work is licensed under CC BY 4.0. To view a copy of this license, visit <https://creativecommons.org/licenses/by/4.0/>

## **Appendix B.** AMSTAR 2 checklist: assessing the methodological quality of systematic reviews.

| 1. **Did the research questions and inclusion criteria for the review include the components of PICO?** | | | |
| --- | --- | --- | --- |
| **For yes:**   - Population - Intervention - Comparator group - Outcome | **Optional (recommended):**   - Timeframe for follow-up | **Final rating:**   - Yes | **Location:**   - Search strategy - Supplemental tables 3-6 |
| 1. **Did the report of the review contain an explicit statement that the review methods were established prior to the conduct of the review and did the report justify any significant deviations from the protocol?** | | | |
| **For partial yes:**  **The authors state that they had a written protocol or guide that included ALL the following**   - Review question(s) - A search strategy - Inclusion/exclusion criteria - A risk of bias assessment | **For yes:**  **As for partial yes, plus the protocol should be registered and should also have specified:**   - A meta-analysis/synthesis plan, if appropriate, *and* - A plan for investigating causes of heterogeneity - Justification for any deviations from the protocol | **Final rating:**   - Yes | **Location:**   - Methods |
| 1. **Did the review authors explain their selection of the study designs for inclusion in the review?** | | | |
| **For yes, the review should satisfy ONE of the following:**   - *Explanation for* including only RCTs - OR *Explanation for* including only NRSI - OR *Explanation for* including both RCTs and NRSI | | **Final rating:**   - Yes | **Location:**   - Search strategy |
| 1. **Did the review authors use a comprehensive literature strategy?** | | | |
| **For partial yes (all the following):**   - Searched at least 2 databases (relevant to research question) - Provided key word and/or search strategy - Justified publication restrictions (e.g., language) | **For yes, should also have (all the following):**   - Searched the reference lists/bibliographies of included studies - Searched trial/study registries - Included/consulted content experts in the field - Where relevant, searched for grey literature - Conducted search within 24 months of completion of the review | **Final rating:**   - Yes | **Location:**   - Search strategy - Supplemental table 2 |
| 1. **Did the review authors perform study selection in duplicate?** | | | |
| **For yes, either ONE of the following:**   - at least two reviewers independently agreed on selection of eligible studies and archived consensus on which studies to include - OR two reviewers selected a sample of eligible studies and achieved good agreement (at least 80%), with the remainder selected by one reviewer | | **Final rating:**   - Yes | **Location:**   - Screening and data extraction |
| 1. **Did the review authors perform data extraction in duplicate?** | | | |
| **For yes, either ONE of the following:**   - At least two reviewers achieved consensus on which data to extract from included studies - OR two reviewers extracted data from a sample of eligible studies and achieved good agreement (at least 80%), with the remainder extracted by one reviewer | | **Final rating:**   - Yes | **Location:**   - Screening and data extraction |
| 1. **Did the review authors provide a list of excluded studies and justify the exclusions?** | | | |
| **For partial yes:**   - Provided a list of all potentially relevant studies that were read in full text form but excluded from review | **For yes, must also have:**   - Justified the exclusion from the review of each potentially relevant study | **Final rating:**   - Yes | **Location:**   - Supplemental tables 8 and 9 |
| 1. **Did the review authors describe the included studies in adequate detail?** | | | |
| **For partial yes (all the following):**   - Described populations - Described interventions - Described comparators - Described outcomes - Described research designs | **For yes, should also have ALL the following:**   - Described population in detail - Described intervention and comparator in details (including doses where relevant) - Described study’s setting - Timeframe for follow-up | **Final rating:**   - Yes | **Location:**   - Search results - Tables 1 and 2 |
| 1. **Did the review authors use a satisfactory technique for assessing the risk of bias (RoB) in individual studies that were included in the review?** | | | |
| **RCTs** | | | |
| **For partial yes, must have assessed RoB from:**   - Unconcealed allocation, *and* - Lack of blinding of patients and assessors when assessing outcomes (unnecessary for objective outcomes such as all-cause mortality) | **For yes, must also have assessed RoB from:**   - Allocation sequence that was not truly random, *and* - Selection of the reported result from among multiple measurements or analyses of a specified outcome | **Final rating:**   - Yes | **Location:**   - Risk of bias - Throughout results - Figure 3 |
| **NRSI** | | | |
| **For partial yes, must have assessed RoB from:**   - Confounding, *and* - Selection bias | **For yes, must also have assessed RoB:**   - Methods used to ascertain exposures and outcomes, *and* - Selection of the reported result from among multiple measurements or analyses of a specified outcome | **Final rating:**   - Yes | **Location:**   - Risk of bias - Throughout results - Figure 3 |
| 1. **Did the review authors report on the sources of funding for the studies included in the review?** | | | |
| **For yes:**   - Must have reported on the sources of funding for individual studies included in the review. Note: Reporting that the reviewers look for this information, but it was not reported by study authors also qualifies | | **Final rating:**   - Yes | **Location:**   - Table 1 footnotes - Table 2 |
| 1. **If meta-analysis was performed did the review authors use appropriate methods for statistical combination of results?** | | | |
| **RCTs** | | | |
| **For yes:**   - The authors justified combining the data in a meta-analysis - AND they used an appropriate weighted technique to combine study results and adjusted for heterogeneity if present - AND investigated the causes of any heterogeneity | | **Final rating:**   - No meta-analysis conducted | **Location:**   - N/A |
| **NRSI** | | | |
| **For yes:**   - The authors justified combining the data in a meta-analysis - AND they used an appropriate weighted technique to combine study results, adjusting for heterogeneity if present - AND they statistically combined effect estimates from NRSI that were adjusted for confounding, rather than combining raw rata, or justified combining raw data when adjusted effect estimates were not available - AND they reported separate summary estimates for RCTs and NRSI separately when both were included in the review | | **Final rating:**   - No meta-analysis conducted | **Location:**   - N/A |
| 1. **If meta-analysis was performed, did the review authors assess the potential impact of RoB in individual studies on the results of the meta-analysis or other evidence synthesis?** | | | |
| **For yes:**   - Included only low risk of bias RCTs - OR, if the pooled estimate was based on RCTs and/or NRSI at variable RoB, the authors performed analyses to investigate possible impact of RoB on summary estimates of effect | | **Final rating:**   - No meta-analysis conducted | **Location:**   - N/A |
| 1. **Did the review authors account for RoB in individual studies when interpreting/discussing the results of the review?** | | | |
| **For yes:**   - Included only low risk of bias RCTs - OR, if RCTs with moderate or high RoB, or NRSI were included the review provided a discussion with the likely impact of RoB on the results | | **Final rating:**   - Yes | **Location:**   - Throughout results and discussion |
| 1. **Did the review authors provide a satisfactory explanation for, and discussion of, any heterogeneity observed in the results of the review?** | | | |
| **For yes:**   - There was no significant heterogeneity in the results - OR if heterogeneity was present the authors performed an investigation of sources of any heterogeneity in the results and discussed the impact of this on the results of the review | | **Final rating:**   - Yes | **Location:**   - Throughout results and discussion |
| 1. **If they performed quantitative synthesis did the review authors carry out an adequate investigation of publication bias (small study bias) and discuss its likely impact on the results of the review?** | | | |
| **For yes:**   - Performed graphical or statistical tests for publication bias and discussed the likelihood and magnitude of impact of publication bias | | **Final rating:**   - No meta-analysis conducted | **Location:**   - N/A |
| 1. **Did the review authors report any potential sources of conflict of interest, including any funding they received for conducting the review?** | | | |
| **For yes:**   - The authors reported no competing interests OR - The authors described their funding sources and how they managed potential conflicts of interest | | **Final rating:**   - Yes | **Location:**   - Title page |

From Shea BJ, Reeves BC, Wells G, et al. Amstar 2: A critical appraisal tool for systematic reviews that include randomised or non-randomised studies of healthcare interventions, or both. *BMJ*. Sep 21 2017;358:j4008. doi:10.1136/bmj.j4008
